# Stress Biology in Cancer: Neuroendocrine–Immune Mechanisms Linking Tumor Progression to Translational Interventions

**DOI:** 10.34133/cancomm.0053

**Published:** 2026-09-22

**Authors:** Chi Ma, Yukang Ma, Xiangling Xing, Chengxi Sun, Li Guan, Helgi B. Schiöth, Wancheng Liu

**Affiliations:** ^1^ Department of Clinical Laboratory, Qilu Hospital of Shandong University, Jinan, Shandong, P. R. China.; ^2^ Department of Radiation Oncology, Qilu Hospital of Shandong University, Jinan, Shandong, P. R. China.; ^3^ Department of Radiation Oncology, Stanford University School of Medicine, Stanford, CA, USA.; ^4^ Department of Surgical Sciences, Functional Pharmacology and Neuroscience, Uppsala University, Uppsala, Sweden.; ^5^ Laboratory of Pharmaceutical Pharmacology, Latvian Institute of Organic Synthesis, Riga, Latvia.

## Abstract

Chronic stress-responsive signaling has emerged as a modifiable dimension of cancer biology by linking systemic neuroendocrine activation to tumor-cell adaptation, metabolic rewiring, immune suppression, tumor microenvironment remodeling, metastatic dissemination, and therapeutic resistance. Persistent activation of the sympathetic nervous system (SNS) and the hypothalamic pituitary adrenal (HPA) axis elevates catecholamines and glucocorticoids, which act through adrenergic receptors and the glucocorticoid receptor (GR) in tumor, stromal, vascular, and immune compartments. These canonical pathways regulate cyclic adenosine monophosphate (cAMP)–protein kinase A (PKA) signaling, GR-dependent transcription, angiogenic and inflammatory programs, extracellular matrix remodeling, myeloid polarization, T cell dysfunction, and resistance to cytotoxic and immune-based therapies. Beyond this classical SNS/HPA framework, emerging evidence indicates that cancer cells also engage noncanonical stress-adaptive hubs, including ion-channel signaling, redox-dependent mitochondrial responses, integrated stress-response pathways, prolactin/prolactin receptor signaling, and neurotrophin-mediated tumor–stroma–nerve crosstalk. These mechanisms are particularly relevant in highly plastic and immunogenic tumors such as melanoma, where stress adaptation intersects with immune visibility, metabolic flexibility, and therapy response. In this review, we synthesized how chronic stress signaling operates across systemic, tumor-intrinsic, immune, stromal, vascular, metabolic, and neural niches. We further mapped cancer-specific mechanisms across major malignancies and evaluated translational strategies, including β- and α-adrenergic blockade, GR modulation, stress-reducing behavioral interventions, and combination approaches with immunotherapy or standard anticancer regimens. Moreover, we discussed clinical challenges, including heterogeneity in stress exposure, context-dependent receptor activity, sex-specific stress biology, evidence maturity, and the need for biomarker-guided patient stratification. This integrated framework supports the development of mechanism-based stress-targeted adjunctive therapies in oncology.

## Introduction

Cancer arises when genetic alterations, environmental exposures, and disrupted regulatory networks erode cellular homeostasis, allowing cells to escape normal growth control, proliferate unchecked, and ultimately disseminate to distant organs [1,2]. As tumors progress, the activation of oncogenes and loss of tumor-suppressor function confer hallmark traits such as sustained proliferative signaling, evasion of programmed cell death, invasion, and metastasis [3,4]. Despite advances in diagnosis and multimodal therapy, relapse, metastatic spread, and treatment resistance remain major clinical challenges, underscoring the need to consider modulatory factors beyond tumor-intrinsic genetics. Stress responses are commonly categorized as acute or chronic. Acute stress, exemplified by the “fight-or-flight” response, supports short-term coping [5,6] and is accompanied by transient surges of catecholamines and glucocorticoids (GCs) that can be adaptive when brief [7]. In contrast, chronic stress reflects sustained work-related, psychological, or environmental hardship and is linked to broad adverse health outcomes, including cardiovascular disease, psychiatric disorders, and cancer [8–14]. Biologically, it is characterized by persistent activation of the sympathetic nervous system (SNS) and the hypothalamic pituitary adrenal (HPA) axis, maintaining elevated catecholamines and GCs—a neuroendocrine pattern frequently observed in patients with cancer at diagnosis and during therapy [15]. These mediators signal through adrenergic receptors and the GC receptor (GR) to engage downstream signaling pathways and transcriptional programs that regulate cellular homeostasis across tissues [16].

**Fig. 1.**
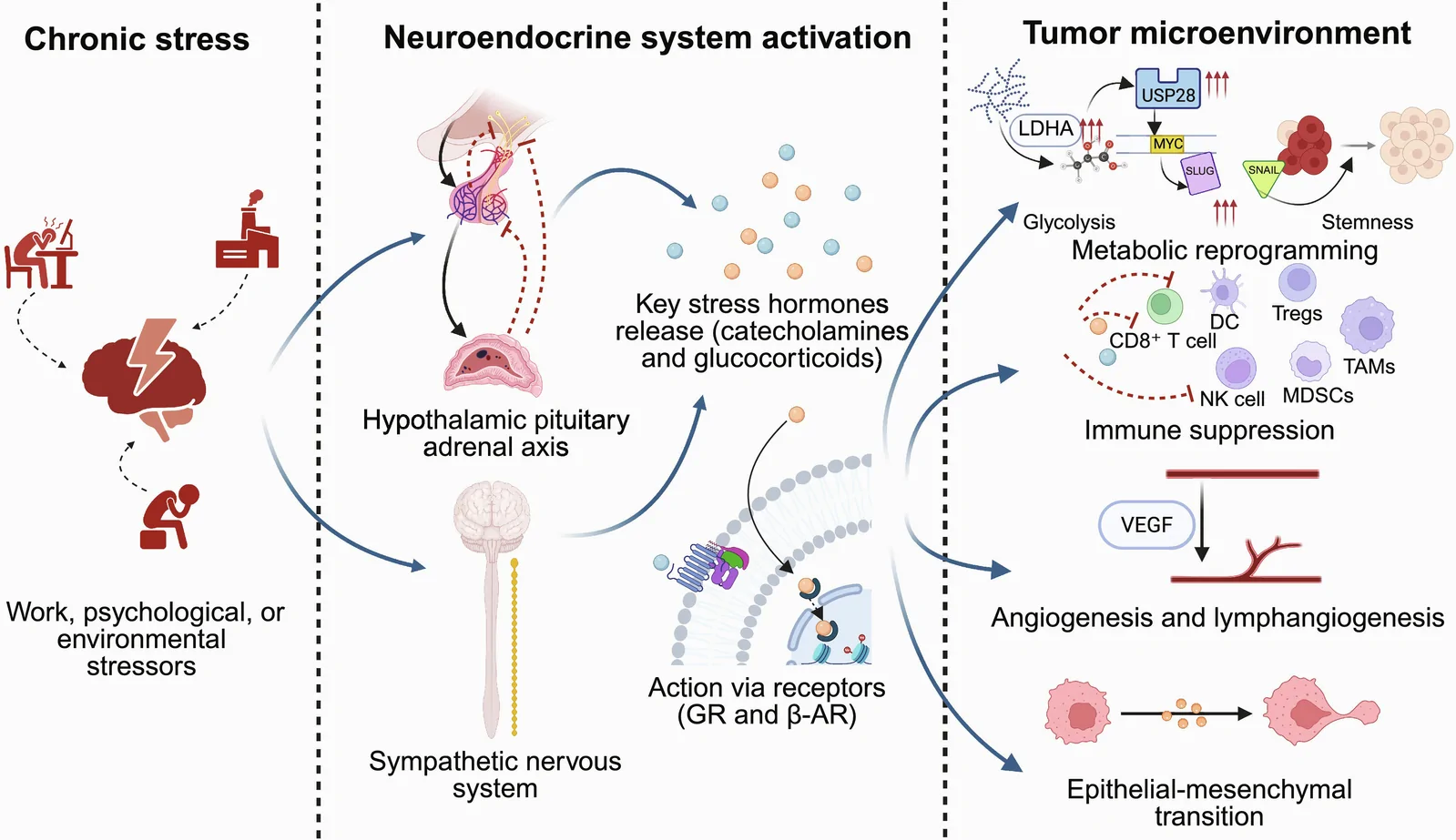
Chronic stress drives neuroendocrine activation and remodels the TME. Chronic psychosocial, work-related, or environmental stressors sustain activation of the SNS and the HPA axis, leading to prolonged release of key stress hormones, including catecholamines and glucocorticoids. These mediators act through β-AR and GR on tumor cells and multiple TME-resident compartments, thereby engaging downstream signaling programs that disrupt cellular homeostasis. Within the TME, chronic stress promotes a tumor-supportive state characterized by metabolic reprogramming (e.g., enhanced glycolytic programs); increased stemness-associated phenotypes; suppressed DC, CD8^+^ T cell, and NK cell function; expanded Tregs, MDSCs, and TAMs; and provascular remodeling, including angiogenesis and lymphangiogenesis (illustrated by VEGF signaling). In parallel, chronic stress facilitates EMT, supporting invasive and metastatic competence. Collectively, this schematic summarizes how sustained SNS/HPA signaling links systemic stress biology to coordinated tumor-intrinsic and microenvironmental changes that favor tumor progression and treatment resistance. This figure was generated with BioRender (https://biorender.com). β-AR, β-adrenergic receptor; DC, dendritic cell; EMT, epithelial–mesenchymal transition; GR, glucocorticoid receptor; HPA, hypothalamic pituitary adrenal axis; LDHA, lactate dehydrogenase A; MDSCs, myeloid-derived suppressor cells; MYC, MYC proto-oncogene, bHLH transcription factor; NK, natural killer cell; SLUG, snail family transcriptional repressor 2 (SNAI2); SNAIL, snail family transcriptional repressor 1 (SNAI1); SNS, sympathetic nervous system; TAM, tumor-associated macrophage; TME, tumor microenvironment; Tregs, regulatory T cells; USP28, ubiquitin-specific peptidase 28; VEGF, vascular endothelial growth factor.

Increasing evidence links chronic stress to cancer outcomes. Sustained elevations of catecholamines and GCs have been associated with tumor initiation, progression, metastasis, and therapy resistance across multiple cancer types [17], and a comprehensive meta-analysis reports associations between stress-related psychosocial exposures and greater cancer incidence, poorer survival, and higher cancer-specific mortality [18]. Consistent with Engel’s view that social and behavioral determinants should be integrated with biological mechanisms, clinical and epidemiologic studies have connected chronic stress, depressive states, and limited social support with cancer progression and, to a lesser extent, initiation [17,19–22]. These observations position chronic stress as a clinically meaningful variable in cancer biology and support efforts to identify actionable stress-related mechanisms for oncology care [16]. Chronic stress acts across systemic, tumor-intrinsic, and microenvironmental levels. Systemically, stress-associated neuroendocrine signaling can alter glucose, lipid, amino acid, and glutamine-related metabolism, thereby creating a metabolic milieu that may support tumor-cell survival and growth [16]. More directly, catecholamines and GCs act through adrenergic receptors and GR on tumor and immune cells within the tumor microenvironment (TME) [16,23–25]. Adrenergic signaling can suppress natural killer (NK) cell and cluster of differentiation 8-positive (CD8^+^) T cell function and expand immunosuppressive myeloid populations, including myeloid-derived suppressor cells (MDSCs) and protumoral macrophages [26–33], whereas GC signaling can further enforce immune tolerance and promote tolerogenic antigen-presenting cell states, particularly in dendritic cells (DCs) [34–40]. These immune alterations converge with local stromal, neural, and vascular stress responses to promote angiogenesis, extracellular matrix (ECM) remodeling, and metastatic competence within the TME (Fig. 1) [23].

Despite these advances, the field faces 2 conceptual challenges. First, chronic stress biology in cancer cannot be fully captured by a linear SNS/HPA hormone cascade; beyond catecholamine- and GC-centered signaling, tumor cells may engage noncanonical stress-adaptive programs and additional mediators that influence redox balance, mitochondrial fitness, immune visibility, tumor–nerve crosstalk, and therapy resistance [41,42]. Second, chronic stress does not act identically across malignancies: Although shared nodes such as β-adrenergic and GR signaling recur, their downstream consequences depend on lineage, receptor context, immune composition, metabolic state, neural niche, and treatment setting. A cancer-specific synthesis is therefore needed to move beyond cataloging experimental observations and to clarify how chronic stress contributes to tumor progression in distinct biological contexts, including highly plastic and immunogenic tumors such as melanoma [43,44].

Chronic stress biology is not only measurable but also, at least in part, modifiable: Behavioral stress-reduction programs and pharmacological modulation of stress signaling may attenuate selected neuroendocrine, immune, and metabolic alterations [45,46]. This supports the development of mechanism-based adjunctive strategies—such as adrenergic blockade, GR modulation, and interventions that lower stress burden—alongside standard cancer therapy. In this review, we synthesized how sustained activation of the SNS and HPA axis, together with emerging noncanonical stress-adaptive and neurotrophic pathways, shapes cancer progression by coordinating tumor-intrinsic programs with metabolic, immune, stromal, vascular, and neural remodeling. We first outlined the biological basis of chronic stress, including canonical and emerging neuroendocrine pathways and sex-dependent features. We then examined stress-associated metabolic reprogramming and receptor-mediated signaling within the TME, and mapped cancer-specific mechanisms across major malignancies. Finally, we evaluated pharmacological and behavioral strategies to mitigate stress-associated tumor aggressiveness and therapy resistance, and discussed challenges and future directions for clinical translation. By distinguishing systemic effects from receptor-mediated local effects and tracing their convergence within cancer-specific TMEs, this review aims to support mechanism-based, biomarker-guided development of stress-targeted adjunctive therapies in oncology.

## Biological Basis of Chronic Stress and Neuroendocrine Signaling

Neuroendocrine signaling provides one of the most consistently characterized mechanistic bridges linking chronic stress to cancer biology [7]. Persistent psychosocial stress sustains activation of the HPA axis and the SNS, resulting in prolonged elevations of GCs and catecholamines. Through GR and β-adrenergic pathways, these mediators can suppress antitumor immunity, rewire tumor metabolism, and promote angiogenesis, collectively favoring tumor growth and metastatic competence [5,7,47–49]. Clinically, chronic psychosocial stress and major depression have been associated with higher recurrence risk and poorer survival in breast cancer (BC), with similar signals reported in ovarian cancer [18,20,50–56]. Observational pharmacology studies further suggest that β-blocker exposure correlates with longer survival across multiple malignancies, although this supports rather than proves a role for sympathetic output in stress-associated tumor progression [57–67]. Importantly, these cascades are context-dependent rather than uniformly tumor-promoting; their direction and magnitude depend on tumor type, receptor subtype, immune composition, sex, stress duration, and timing relative to treatment. The following sections summarize canonical HPA/GR and SNS/β-adrenergic signaling, stress-driven immune remodeling, sex-dependent variation in stress responses, and emerging stress-adaptive or neurotrophic pathways that extend the classical neuroendocrine framework.

### HPA axis: GC–GR signaling

Under chronic stress, HPA axis activity remains elevated, leading to persistently increased circulating GCs [68]. GCs act through GR in both tumor and immune cells, producing broad effects that include metabolic reprogramming, immune suppression, and proangiogenic signaling [68,69]. At the tumor-cell level, GR activation induces transcriptional programs that enhance anti-apoptotic capacity, reduce chemotherapy-induced apoptosis, and support tumor survival and growth [70–72]. Accordingly, experimental models show that prolonged stress exposure or exogenous GC treatment can increase tumor burden and promote paclitaxel resistance [73]. In BC models that are GR-positive but estrogen receptor (ER)-, progesterone receptor (PR)-, and human epidermal growth factor receptor 2 (HER2)-negative, GR blockade enhances paclitaxel cytotoxicity and increases apoptosis, indicating that GR activity can function as a modulator of therapy response in defined molecular contexts [74]. These findings position the GC–GR axis as a grounded and potentially druggable link between chronic stress biology and treatment resistance [68–74]. GC–GR signaling should therefore be interpreted as context-dependent rather than uniformly tumor-promoting. In lymphoid malignancies, including acute lymphoblastic leukemia (ALL), lymphoma, and multiple myeloma, GCs can induce apoptosis and remain a therapeutic mainstay, whereas in many solid epithelial tumors, GR activation is more often linked to survival programs, chemotherapy resistance, and metastatic competence [75,76]. Thus, the net effect of GC–GR signaling depends on tumor lineage, GR isoform and co-regulator context, and the dose and duration of exposure [75]. Importantly, GR activation is not stress-specific, because the same receptor can be engaged by stress-induced adrenal GCs, exogenous therapeutic steroids, or GCs generated locally within tumors through intratumoral steroid metabolism, including 11β-hydroxysteroid dehydrogenase type 1 (11β-HSD1)-mediated regeneration of active cortisol or corticosterone [77,78]. Cancer cells and tumor-infiltrating immune or stromal compartments can therefore sustain GR signaling in stress-independent settings, as shown by cancer-associated GC systems and GR transcriptional activity in BC and other tumor models [77–79]. Accordingly, GR-attributable tumor effects should not automatically be attributed to chronic stress. Causal attribution to stress requires evidence that a defined stressor increases endogenous GC signaling and that blockade of the stress axis, GR, or local GC regeneration reverses the tumor phenotype. Moreover, while HPA/GR signaling provides a direct route by which chronic stress can alter tumor-cell survival programs and therapeutic sensitivity, chronic stress also persistently engages the SNS, which often exerts prominent effects on stromal/vascular remodeling and metastatic spread through β-adrenergic signaling.

### SNS: Catecholamines and β-adrenergic signaling

Chronic stress maintains SNS activation and elevates catecholamine levels, enabling sustained stimulation of β-adrenergic receptors on tumor cells and stromal components. β2-Adrenergic receptors (β2-AR) are most often the dominant subtype, although β1- or β3-AR can predominate in particular tumor types. Downstream cyclic adenosine monophosphate (cAMP)–protein kinase A (PKA) signaling promotes proangiogenic, inflammatory, and ECM-remodeling programs, thereby fostering a prometastatic TME [80–82]. In an orthotopic syngeneic mouse model of BC, restraint stress had little effect on primary tumor growth but increased metastasis to the lymph nodes and lungs by approximately 30-fold [83]. Direct causal preclinical evidence links stress and stress hormones to metastasis: Restraint stress or β-adrenergic stimulation increases metastatic dissemination, whereas β-blockade with propranolol, chemical sympathectomy with 6-hydroxydopamine (6-OHDA), or other interruption of sympathetic signaling reduces stress-enhanced metastasis, supporting causality rather than mere correlation [81,82,84]. GC signaling may contribute in parallel in selected tumor contexts, but human evidence remains largely associative, including β-blocker pharmacoepidemiology and cortisol-rhythm survival studies [57,85]. A key mechanistic theme is stromal and vascular remodeling: Chronic stress can alter lymphatic and blood vessels and recruit macrophages, lowering physical and immunologic barriers to tumor-cell escape from the primary site [82,84,86,87]. Consistent with this model, pharmacological blockade of sympathetic/β-adrenergic signaling prevents stress-induced lymphatic remodeling and reduces lymph-node metastasis in vivo. These preclinical findings align with convergent clinical observations in BC patients, supporting the further investigation of sympathetic dampening as a plausible translational strategy [82,84,87]. Although systemic β-blockers act on both tumor and stromal compartments and therefore cannot fully disentangle cell-type-specific targets, the overall evidence supports SNS/β-adrenergic output as a druggable axis linking chronic stress to metastatic spread [84,87,88]. The benefit of targeting this axis is, moreover, time-dependent: The perioperative period constitutes a discrete, high-risk window in which surgery-evoked surges of catecholamines and prostaglandins transiently amplify metastatic seeding, making the timing of intervention itself a determinant of benefit. Consistent with this, perioperative β-adrenergic blockade combined with cyclooxygenase-2 (COX-2) inhibition improved prometastatic biomarkers in a phase II BC trial [83] and improved survival in 2 preclinical models of spontaneous postoperative metastasis in mice [89]. Importantly, HPA- and SNS-derived mediators do not act solely through tumor-intrinsic signaling. A substantial portion of their tumor-promoting impact is mediated through coordinated immunologic changes, a mechanistic domain classically situated within the framework of psychoneuroimmunology.

### Psychoneuroimmunology: Stress-driven immune suppression and myeloid remodeling

Chronic stress alters both the magnitude and temporal patterning of hormone secretion and is broadly immunosuppressive [90–92]. Through GC signaling, chronic stress can reduce cytokine gene expression, synthesis, and release, thereby dampening antitumor immune responses [90–92]. Disruption of normal cortisol circadian rhythm has been associated with BC onset and disease course [84,93]; in metastatic settings, flattened diurnal cortisol patterns coincide with reduced numbers and cytotoxic function of NK cells and predict earlier mortality, implicating NK cells as candidate effectors and biomarkers of stress-associated cancer outcomes [68,85]. In parallel, β-adrenergic activation suppresses lymphocyte activity, reduces NK cytotoxicity, and weakens DC antigen presentation, thereby further limiting effective antitumor immunity [68]. Beyond lymphoid suppression, chronic psychosocial stress is also associated with dysregulated inflammatory signaling, including increased production of proinflammatory mediators that may foster a tumor-promoting immune microenvironment [94]. Together, these observations support a model in which chronic stress reconfigures immune surveillance through both GC–GR endocrine signaling and adrenergic pathways, shifting the tumor ecosystem toward immune evasion and dissemination. Clinical associations between psychological stress or depression and cancer stage at diagnosis or histopathological grade remain limited and inconsistent, partly because distress may reflect delayed help-seeking or screening, treatment adherence, or reverse causation from advanced disease rather than a direct stress-driven tumor effect [95,96]. A more consistent stratification signal may be molecular rather than purely psychosocial; for example, tumoral β2-AR expression has been associated with advanced tumor–node–metastasis (TNM) stage, poor differentiation, and nodal metastasis in gastric cancer [97]. Sex- and stage-stratified clinical studies that pair psychosocial measures with neuroendocrine biomarkers, receptor expression, and histopathologic features are therefore needed.

### Sex differences in the stress response and their relevance to cancer

HPA-axis responses to stress are sexually dimorphic, making sex an important biological variable when considering neuroendocrine stress signaling [98,99]. At the HPA axis level, sex steroids modulate corticotropin-releasing hormone (CRH), adrenocorticotropic hormone, and downstream GC output, with estrogens generally tending to potentiate HPA reactivity and androgens tending to restrain it, although these effects remain context-dependent [98,99]. Basal and stress-induced cortisol or corticosterone levels differ by sex and can fluctuate with reproductive hormonal status, including across the menstrual or estrous cycle [98,99]. In humans, cortisol responses are also stressor-type dependent, with men showing stronger responses to achievement-oriented stressors and women showing stronger responses to social-rejection stressors in some experimental settings, which is relevant when psychosocial stress exposure is modeled or measured in cancer cohorts [100]. Sex differences also extend to SNS and catecholamine biology, including sympatho-adrenal activation, catecholamine release and clearance, and adrenergic responsiveness, all of which are directly relevant to β-adrenergic signaling, including β2-AR (encoded by *ADRB2*), in tumor cells and the TME [101]. In parallel, sex differences in GR expression on circulating leukocyte subsets and in GC responsiveness indicate that stress-driven immunosuppression may produce different effects on dendritic-cell priming, CD8^+^ T cell function, NK cell cytotoxicity (NKCC), and myeloid polarization across sexes [102,103]. This is relevant to cancer because many nonreproductive malignancies show male-biased incidence and/or mortality, while sex-biased tumor immunity, inflammation, metabolism, and treatment responses are increasingly recognized across cancer types [104,105]. Within this framework, sex differences in stress signaling could plausibly contribute to sex-biased tumor progression by altering the balance between adrenergic tumor-promoting pathways, GR-dependent immune suppression, and antitumor effector immunity, although this possibility remains underexplored in stress–cancer studies [103–105]. In hormone-related cancers, the interaction may be more direct because breast, prostate, and ovarian cancers are shaped by estrogenic or androgenic programs that can intersect with stress-responsive β-adrenergic, GR, metabolic, and immune pathways [104,105]. For example, estrogen or ER signaling may interact with β2-AR signaling to influence BC progression, immune remodeling, and therapeutic resistance, whereas androgen receptor signaling may interact with adrenergic and GC pathways during stress-associated prostate cancer progression [104,105]. Methodologically, many preclinical biomedical studies have historically relied on single-sex designs or have not analyzed both sexes separately, and stress–oncology models are particularly vulnerable to this limitation because many paradigms use sex-restricted breast, ovarian, or prostate cancer systems [106]. Moreover, the available clinical evidence on β-blockers and cancer survival is heterogeneous and has generally not been analyzed by patient sex, having instead been examined in relation to factors such as tumor type, β-blocker selectivity, and cancer stage [107]. Therefore, sex should be treated as a biological variable in both preclinical and clinical stress–cancer research, with sex-stratified analyses of neuroendocrine readouts, immune remodeling, metabolic adaptation, metastatic endpoints, and responses to β-adrenergic or GR-modulating interventions. Integrating sex as a mechanistic modifier will strengthen the neuroendocrine–immune framework outlined above and help clarify when chronic stress signaling is most likely to drive tumor progression or represent a therapeutically actionable vulnerability.

Collectively, the HPA/GC–GR axis, SNS/β-adrenergic signaling, and downstream immunologic remodeling form an integrated neuroendocrine–immune network through which chronic stress can influence cancer progression and therapeutic vulnerability, with sex acting as a biological modifier of these pathways. This framework establishes a mechanistic rationale for targeting stress signaling as a modifiable dimension of cancer biology. It also motivates deeper examination of how these pathways converge on specific tumor hallmarks—such as metabolic rewiring, angiogenic and lymphatic remodeling, and pre-metastatic niche formation—and how they may be exploited for biomarker-guided adjunct interventions.

### Emerging stress-adaptive signaling pathways beyond classical neuroendocrine signaling

Although chronic stress in cancer has classically been framed around sustained activation of the SNS and the HPA axis, tumor stress adaptation cannot be fully explained by catecholamine- and GC-centered pathways alone. These canonical mediators provide important systemic and receptor-mediated inputs, but cancer cells also interpret stressful conditions through tumor-intrinsic programs that sense membrane perturbation, oxidative pressure, mitochondrial dysfunction, nutrient limitation, and translational stress [41,108,109]. Rather than replacing the SNS/HPA framework, these noncanonical pathways extend it by explaining how systemic stress contexts may be converted into tumor-cell fate decisions, including survival, phenotypic plasticity, therapy resistance, immune invisibility, or immune-mediated elimination. This expanded view is particularly relevant to melanoma and other highly adaptive tumors, in which metastatic competence and immune escape depend strongly on stress tolerance [41–44].

Ion-channel-mediated stress sensing illustrates this additional layer of regulation. A recent melanoma study identified transient receptor potential melastatin 8 (TRPM8) as a noncanonical vulnerability in metastatic melanoma [108]. Pharmacological modulation of TRPM8 induced calcium-independent mitochondrial apoptosis, accompanied by reactive oxygen species (ROS) accumulation, mitochondrial membrane depolarization, cytochrome c release, caspase-3 activation, ataxia-telangiectasia mutated (ATM)/p53 pathway engagement, and induction of proapoptotic proteins. Importantly, TRPM8 modulation also increased expression of the NK cell-activating ligand ULBP1, thereby enhancing melanoma-cell susceptibility to NK cell-mediated cytotoxicity through the ULBP1/NKG2D axis [42,110]. This mechanism links membrane-associated signaling, redox stress, mitochondrial injury, immune visibility, and innate immune surveillance in a single axis. However, the finding should be interpreted cautiously: It does not demonstrate that chronic psychological stress directly signals through TRPM8 but shows that melanoma cells possess noncanonical stress-adaptive circuits whose perturbation can simultaneously trigger mitochondrial cell death and increase immune recognition [42].

ROS-dependent signaling and mitochondrial adaptation provide the mechanistic context for this observation. In melanoma, oxidative stress functions both as a barrier to metastasis and as a selective pressure that favors stress-tolerant clones. Cells that successfully disseminate can acquire reversible metabolic programs that improve resistance to oxidative damage, including increased reliance on nicotinamide adenine dinucleotide phosphate (NADPH)-generating pathways [43]. This supports a threshold model: Compensated ROS may promote adaptation, dissemination, and survival, whereas excessive or poorly buffered mitochondrial ROS can drive apoptotic collapse. The TRPM8–ROS–mitochondrial apoptosis axis exemplifies the latter condition, in which redox stress exceeds adaptive capacity and becomes linked not only to tumor-cell death but also to enhanced NK cell recognition.

The integrated stress response represents another noncanonical bridge between tumor-cell adaptation and immune evasion. The ISR is activated by amino acid deprivation, proteotoxic stress, mitochondrial dysfunction, and related adverse conditions, converging on eIF2α phosphorylation and selective ATF4-dependent transcription. Recent evidence indicates that the ISR–ATF4 axis can promote immune evasion through lipocalin 2 (LCN2), which favors immunosuppressive macrophage infiltration, limits CD8-positive T cell accumulation, and contributes to T cell exclusion [41]. This immune-dependent phenotype has also been observed in syngeneic models including B16F10 melanoma [41]. Collectively, these data support a multilayered model of stress biology in cancer, in which classical neuroendocrine signals intersect with ion-channel programs, redox thresholds, mitochondrial stress responses, and ISR-dependent immunosuppressive outputs to determine whether stressed tumor cells adapt, die, or become visible to antitumor immunity.

### Additional stress-responsive neuroendocrine and neurotrophic mediators

Beyond catecholamines and GCs, additional stress-responsive mediators may broaden the neuroendocrine framework, although their evidence base is generally more context-dependent than that for canonical SNS/HPA signaling. Prolactin is a pleiotropic peptide hormone regulated by tonic dopaminergic inhibition and stress- and sleep-related physiological states [111]. Through the prolactin receptor (PRLR), a class I cytokine receptor expressed across epithelial and immune compartments, prolactin activates Janus kinase 2 (JAK2)–signal transducer and activator of transcription 5 (STAT5) and can also engage mitogen-activated protein kinase (MAPK)/extracellular signal-regulated kinase (ERK), Src-family, and phosphoinositide 3-kinase (PI3K)/protein kinase B (AKT)/mechanistic target of rapamycin (mTOR)-related signaling [112]. In selected cancers, prolactin/PRLR signaling has been associated with proliferation, survival, stemness, invasion, and therapy resistance [112,113], while its cytokine-like activity may influence antigen presentation, cytokine production, T cell activation, regulatory T cell (Treg) function, macrophage behavior, and NK cell responses [114]. These effects can extend to TME remodeling, as shown by prolactin/PRLR-associated stromal–epithelial crosstalk and fibrosis in pancreatic ductal adenocarcinoma [115]. However, direct evidence for a chronic psychological stress–prolactin–cancer progression causal axis remains less mature than that for catecholamines and GCs. Prolactin should therefore be framed as a plausible, context-dependent modifier rather than an established causal stress–cancer effector [116,117].

Neurotrophin signaling represents a mechanistically distinct neural–tumor niche axis rather than simply another growth-factor pathway. During stress-associated neural remodeling, tumor cells, cancer-associated fibroblasts (CAFs), and neural elements can produce nerve growth factor (NGF), brain-derived neurotrophic factor (BDNF), and related ligands that signal through Trk receptors and p75NTR, with Trk activation engaging Ras/MAPK, PI3K/AKT, and phospholipase Cγ (PLCγ) programs involved in survival, motility, neurite outgrowth, and stromal remodeling [118–120]. In prostate cancer, proNGF is linked to high-grade disease and nerve infiltration, while patient-derived CAF studies identify a reciprocal NGF–TrkA circuit among tumor cells, CAFs, and neural elements that promotes tumor growth, epithelial plasticity, and perineural invasion [121,122]. A complementary YAP1/TEAD1–NGF–CCL2/CCR2 feedback loop further connects CAF-driven neural recruitment with epithelial–mesenchymal transition (EMT) and perineural spread [123]. In BC, nerve fiber infiltration has been associated with tumor NGF expression and lymph-node invasion; norepinephrine/β2-adrenergic signaling increases NGF production in triple-negative BC; and NGF/TrkA or proNGF–TrkA/EphA2–Src signaling supports migration, invasion, spheroid growth, matrix remodeling, blood–brain barrier transmigration, and brain metastatic colonization [124–126]. Thus, neurotrophins should be discussed as context-dependent mediators of tumor–stroma–nerve crosstalk that may intersect with adrenergic stress signaling, rather than as universally established causal outputs of chronic psychological stress [120].

## Systemic and Tumor-Intrinsic Metabolic Reprogramming under Chronic Stress

Tumor metabolism denotes the distinctive metabolic state of cancer cells, which supports energy production, biosynthetic precursor supply, and redox homeostasis to sustain rapid growth and survival [127]. Across prostate, breast, gastric, lung, and skin cancers, converging evidence indicates that chronic stress can initiate tumorigenesis and accelerate disease progression [12,81,97,128–136]. Importantly, the metabolic consequences of chronic stress should be interpreted in relation to cancer-relevant phenotypes rather than as isolated systemic stress responses. In tumor-bearing hosts, stress-associated neuroendocrine signaling may reshape glucose-, lipid-, amino acid-, and glutamine-related metabolism in ways that promote tumor-cell adaptation, remodel the TME, impair antitumor immunity, and influence therapeutic response [137–139]. In parallel, cancer cells undergo extensive metabolic rewiring, including enhanced aerobic glycolysis, increased synthesis of fatty acids and other lipids, and glutamine-driven anaplerosis. These adaptations are regulated by key signaling pathways such as PI3K, hypoxia-inducible factors (HIFs), p53, MYC proto-oncogene, bHLH transcription factor (MYC), and the AMP-activated protein kinase (AMPK)–liver kinase B1 (LKB1) axis [140–142]. Based on this framework, the following sections focus on stress-associated metabolic alterations with direct or mechanistically plausible relevance to tumor growth, metastatic dissemination, immune suppression, and therapy resistance, while broader stress-associated physiological findings are discussed only when they help explain cancer-associated metabolic remodeling.

### Glucose metabolism

Chronic stress influences glucose metabolism in cancer primarily by enhancing tumor glycolytic reprogramming rather than merely disrupting systemic glucose homeostasis. In tumor-bearing models, chronic stress-associated depression-like states exacerbate tumor progression and metastatic dissemination in BC and lung cancer (LC), and this effect is accompanied by selective amplification of aerobic glycolysis within tumor tissues [143]. Specifically, chronic stress increases glycolytic metabolites and up-regulates key glycolytic enzymes, including hexokinase 2 (HK2), phosphofructokinase, platelet type (PFKP), pyruvate kinase M2 (PKM2), and lactate dehydrogenase A (LDHA), consistent with reinforcement of a Warburg-like metabolic program that supports tumor growth and survival [143].

**Fig. 2.**
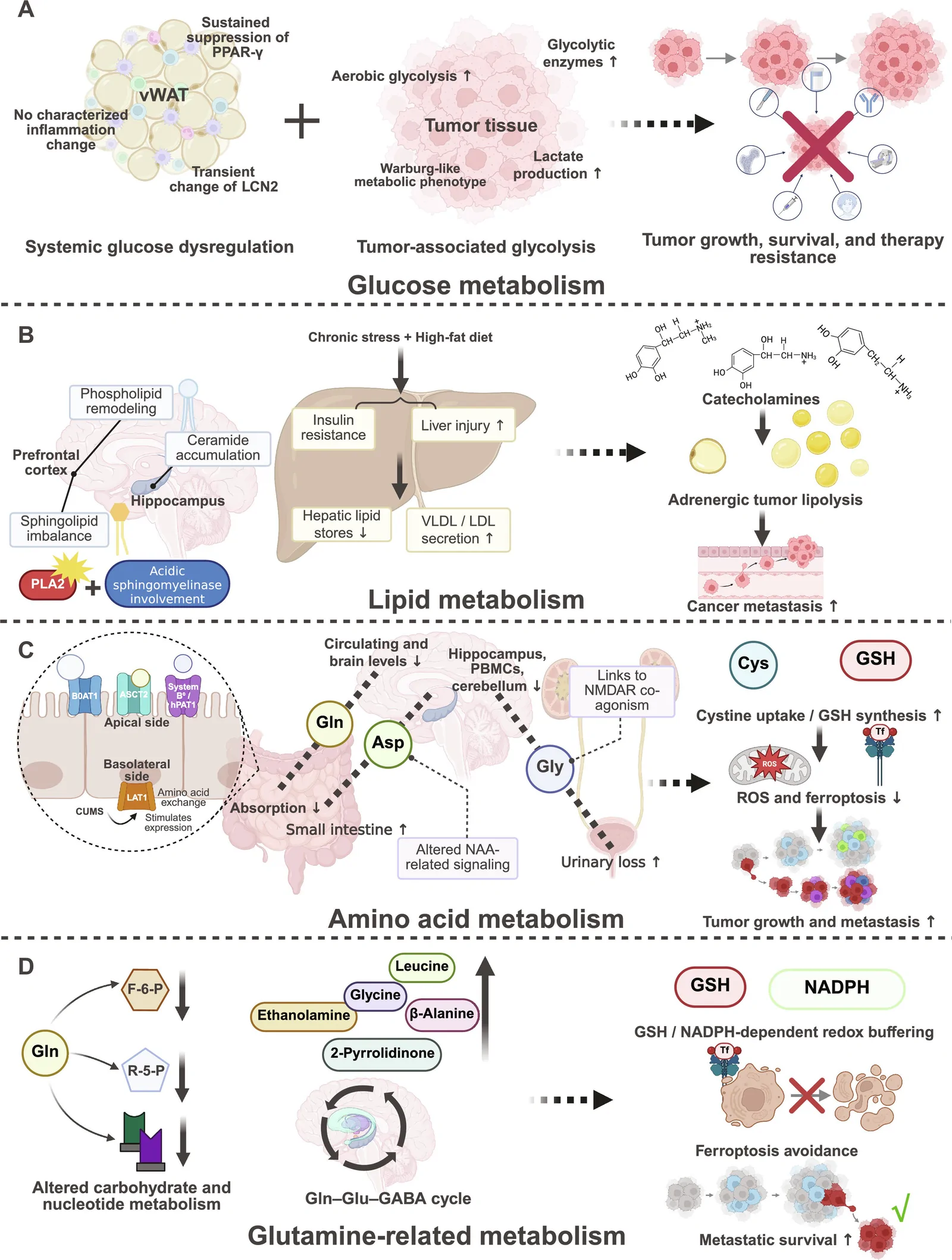
Chronic stress-associated metabolic remodeling with tumor-relevant outputs. (A) Chronic stress induces systemic glucose dysregulation, including sustained suppression of PPAR-γ in vWATs and transient LCN2-linked changes, while preferentially enhancing tumor-associated aerobic glycolysis. Increased glycolytic enzyme activity and lactate production support a Warburg-like metabolic phenotype, thereby contributing to tumor growth, survival, and therapy resistance. (B) Chronic stress remodels lipid metabolism through systemic and tumor-intrinsic routes. Brain and hepatic lipid alterations, including phospholipid remodeling, sphingolipid imbalance, ceramide accumulation, insulin resistance, altered hepatic lipid storage, and increased VLDL/LDL secretion, represent systemic metabolic correlates of stress exposure. In parallel, catecholamine-driven adrenergic signaling can promote tumor lipolysis, linking stress-associated lipid mobilization to metastatic progression. (C) Chronic stress alters amino acid handling through intestinal transport and systemic amino acid redistribution, affecting stress-sensitive amino acids such as glutamine, glycine, and aspartate. In tumor-relevant contexts, altered amino acid availability can support cystine uptake and GSH synthesis, thereby reducing ROS accumulation and ferroptosis and promoting tumor growth and metastasis. (D) Chronic stress perturbs glutamine-related metabolic networks, including carbohydrate and nucleotide metabolism and the glutamine–glutamate–GABA cycle. From a tumor-focused perspective, glutamine-linked metabolism may support GSH- and NADPH-dependent redox buffering, ferroptosis avoidance, and metastatic survival. Together, these panels illustrate how chronic stress-associated systemic metabolic remodeling can converge with tumor-intrinsic metabolic adaptation to favor cancer progression and therapeutic resistance. This figure was generated with BioRender (https://biorender.com). ASCT2, alanine–serine–cysteine transporter 2; Asp, aspartate; B^0^AT1, broad neutral amino acid transporter 1; CUMS, chronic unpredictable mild stress; Cys, cysteine; F-6-P, fructose 6-phosphate; GABA, γ-aminobutyric acid; Gln, glutamine; Glu, glutamate; Gly, glycine; GSH, glutathione; hPAT1, human proton-coupled amino acid transporter 1; LAT1, L-type amino acid transporter 1; LCN2, lipocalin 2; LDL, low-density lipoprotein; NAA, *N*-acetylaspartate; NADPH, reduced nicotinamide adenine dinucleotide phosphate; NMDAR, *N*-methyl-d-aspartate receptor; PBMCs, peripheral blood mononuclear cells; PLA2, phospholipase A2; PPAR-γ, peroxisome proliferator-activated receptor γ; R-5-P, ribose 5-phosphate; ROS, reactive oxygen species; Tf, transferrin; VLDL, very-low-density lipoprotein; vWAT, visceral white adipose tissue.

Systemically, chronic stress can also induce persistent glucose intolerance and hyperinsulinemia even after circulating corticosterone levels normalize [144]. This phenotype is associated with durable suppression of peroxisome proliferator-activated receptor γ (PPAR-γ) in visceral white adipose tissue (vWAT) and LCN2-linked inflammatory remodeling, as summarized in Fig. 2A [144]. However, in the context of cancer progression, the major relevance of these systemic changes is that they may provide a permissive metabolic environment in which tumor cells more readily exploit glycolytic and anabolic programs.

Mechanistically, colorectal cancer (CRC) provides direct evidence that stress-associated catecholamine signaling promotes tumor glycolysis and growth. Chronic stress facilitates CRC tumor growth in vivo, while epinephrine promotes CRC cell proliferation in vitro [137]. Stressed tumors show elevated glycolytic intermediates, and stressed or epinephrine-treated CRC cells exhibit increased glucose consumption, lactate production, and extracellular acidification rate (ECAR) [137]. At the molecular level, catecholamine signaling activates a β2-AR–cAMP–PKA axis that engages cAMP response element-binding protein 1 (CREB1), leading to the transcriptional induction of glycolysis-associated genes such as glucose transporter 1 (GLUT1), HK2, and PFKP [137]. Functionally, inhibition of glycolysis with 2-deoxy-d-glucose (2-DG) or blockade of β2-AR signaling with ICI-118,551 reverses stress-enhanced CRC proliferation and suppresses stress-associated tumor growth [137]. Together, these findings support a cancer-focused model in which chronic stress promotes tumor progression by reinforcing glycolytic metabolism, thereby supporting proliferation, survival, and metastatic competence.

### Lipid metabolism

Chronic stress can also promote tumor progression by engaging lipid-mobilizing pathways that support metastatic dissemination. In BC, chronic stress-associated epinephrine signaling has been mechanistically linked to tumor lipolysis and lung metastasis [139]. Circulating epinephrine levels positively correlate with tumoral ubiquitin-specific peptidase 22 (USP22) expression, and epinephrine stabilizes USP22 through AKT-mediated phosphorylation [139]. Stabilized USP22 deubiquitinates and stabilizes forkhead box O1 (FOXO1), thereby enhancing FOXO1-dependent transcription of adipose triglyceride lipase (ATGL). This pathway promotes ATGL-mediated lipolysis, reflected by reduced triacylglycerol (TAG) and increased diacylglycerol (DAG) and free fatty acids, and facilitates metastatic progression [139]. Deletion of USP22 abrogates epinephrine-induced ATGL up-regulation and lipolysis and suppresses epinephrine-driven BC lung metastasis [139]. Moreover, pharmacological USP22 inhibition synergized with β-adrenergic blockade to reduce tumor growth and lung metastasis in BC cell and mouse models [139]. These findings provide a direct mechanistic connection between chronic stress, adrenergic signaling, tumor lipid utilization, and metastatic dissemination. Beyond tumors, chronic stress also remodels lipid metabolism in central and peripheral tissues, including GC-linked phospholipid and sphingolipid changes in stress-sensitive brain regions and altered hepatic lipid handling under dietary stress, as summarized in Fig. 2B [145–149]. These systemic lipid changes may influence lipid substrate availability and inflammatory tone, but their relevance to cancer progression remains less direct than tumor-intrinsic adrenergic lipolysis pathways. Therefore, the most cancer-relevant evidence currently supports a model in which chronic stress promotes metastatic competence by activating adrenergic lipid-mobilization circuits within tumor cells, particularly the AKT–USP22–FOXO1–ATGL axis in BC.

### Amino acid metabolism

Amino acid metabolism is highly relevant to cancer progression because malignant cells depend on amino acid uptake and utilization to sustain biosynthesis, redox control, immune evasion, and adaptation to metabolic stress. In the context of chronic stress, the most cancer-relevant question is therefore not only whether stress alters systemic amino acid handling, but whether these alterations translate into tumor-supportive metabolic programs. Current evidence suggests 2 potential routes: systemic remodeling of amino acid availability and tumor-intrinsic stress-hormone signaling that directly rewires amino acid-linked redox defenses. The clearest direct tumor-focused evidence comes from liver cancer models exposed to chronic restraint stress. Multi-omics profiling links chronic stress to broad amino acid metabolic reprogramming, including cysteine/methionine metabolism, alanine, aspartate, and glutamate metabolism, and arginine biosynthesis, together with up-regulation of alanyl aminopeptidase (ANPEP/CD13) in tumors [150]. Mechanistically, chronic stress-induced GCs promote nuclear translocation of nuclear receptor subfamily 3 group C member 1 (NR3C1) and transcriptional activation of ANPEP. ANPEP then stabilizes solute carrier family 3 member 2 (SLC3A2) by blocking membrane-associated RING-CH-type finger 8 (MARCH8)-mediated lysosome-dependent degradation, thereby enhancing intracellular l-cystine import, increasing glutathione (GSH) synthesis, suppressing ROS-driven lipid peroxidation and ferroptosis, and ultimately supporting liver cancer growth and metastasis under chronic stress [150]. This mechanism provides a direct link between chronic stress, amino acid transport, redox buffering, ferroptosis resistance, and tumor progression. By contrast, evidence on chronic stress-associated intestinal amino acid transport is more indirect for cancer. Chronic stress can alter amino acid handling along the gut–brain axis through several intestinal and neutral amino acid transporters, as summarized in Fig. 2C [151–161]. These changes may influence systemic availability of amino acids such as glutamine and glycine, and some of these transporters (e.g., LAT1 and ASCT2) are also implicated in cancer-related amino acid uptake [155,161]. However, the direct contribution of stress-induced intestinal transporter remodeling to tumor growth remains insufficiently established. Therefore, this axis should be interpreted as a potential systemic conditioning mechanism rather than as a proven direct driver of tumor progression.

Overall, chronic stress may affect tumor amino acid metabolism through both host-level changes in amino acid availability and more direct tumor-intrinsic redox programs. Among the currently discussed pathways, the GC–NR3C1–ANPEP–SLC3A2 axis provides the strongest mechanistic evidence linking chronic stress to amino acid-dependent tumor growth and metastasis, whereas gut–brain amino acid transport remains a plausible but less directly validated contributor that requires tumor-bearing stress models and metabolic tracing for confirmation.

### Glutamine-related metabolism

Glutamine metabolism is a central axis of cancer metabolic reprogramming. In many tumors, glutamine supports tricarboxylic acid (TCA) cycle anaplerosis, nucleotide and amino acid biosynthesis, GSH production, and ROS control, thereby helping malignant cells maintain proliferation and survive metabolic and therapeutic stress [162]. From a cancer-focused perspective, the key issue is whether chronic psychological stress directly rewires intratumoral glutamine utilization or instead affects glutamine-related networks indirectly through systemic metabolic, neuroendocrine, and immune remodeling.

Chronic stress perturbs glutamine-related metabolism in stress-sensitive brain regions, including alterations in the glutamine–glutamate–γ-aminobutyric acid (GABA) cycle, as summarized in Fig. 2D [163–165]. However, these central nervous system (CNS)-centered findings do not establish direct reprogramming of intratumoral glutamine metabolism. Direct evidence that chronic psychological stress alters intratumoral glutamine flux remains limited. Accordingly, the tumor relevance of glutamine-related stress biology should be framed with appropriate caution. A functional intersection between chronic stress and tumor glutamine dependence is biologically plausible because chronic stress alters neuroendocrine signaling, immune function, systemic nutrient availability, and redox pressure, all of which can influence tumor metabolic adaptation. Future studies should therefore combine tumor-bearing chronic stress models with isotope tracing, single-cell or spatial metabolic profiling, and assessment of glutamine transporters and GSH-linked redox pathways in tumor and immune compartments. This would clarify whether stress-associated glutamine remodeling is a causal driver of tumor growth and therapy resistance or primarily a systemic correlate of stress physiology.

Melanoma further illustrates how redox-linked metabolic plasticity can determine metastatic competence during dissemination [43,44,166,167]. Although these melanoma studies do not establish chronic psychological stress as the upstream driver, they provide tumor-focused models for understanding how cancer cells survive oxidative and ferroptotic pressures during metastatic transit. Human melanoma cells entering the blood and visceral organs experience oxidative stress that limits distant metastasis, whereas successfully metastasizing cells undergo reversible metabolic adaptations that enhance antioxidant capacity, including increased dependence on NADPH-generating enzymes in the folate pathway [43]. In parallel, the lymphatic niche protects disseminating melanoma cells from oxidative stress and ferroptosis, partly through a metabolite environment characterized by higher GSH and oleic acid and lower free iron, thereby increasing survival during subsequent blood-borne spread [44]. Recent evidence further extends this redox-resistance paradigm by showing that FTSJ1-dependent selenocysteine tRNA methylation supports antioxidant selenoprotein translation, oxidative-stress resistance, and melanoma metastatic colonization [166]. Together with stress-linked amino acid and GSH pathways discussed above, these melanoma findings emphasize that metabolic rewiring is not limited to fuel utilization but also includes redox buffering, ferroptosis avoidance, and niche-dependent survival. Melanoma can therefore serve as a particularly informative model for testing whether neuroendocrine stress signaling converges with tumor-intrinsic redox programs to support metastatic adaptation.

## Chronic Stress Signaling and Remodeling of the TME

The TME is a dynamic ecosystem composed of malignant cells, stromal and immune populations, ECM, soluble mediators (e.g., cytokines), and the tumor vasculature [168,169]. Metabolic reprogramming within this milieu supplies energy and biosynthetic precursors to support rapid proliferation and survival under genetic and environmental stress [170]. Importantly, TME states and cancer metabolism interact bidirectionally: Metabolic changes shape local immunity, stromal behavior, and vascular function, whereas TME cell populations impose metabolic pressures on cancer cells [171,172]. Against this background, chronic stress emerges as a systemic modifier of TME composition and function, associating with altered immune-cell states, cytokine programs, angiogenesis, EMT, and matrix damage [16].

### Mechanistic basis: How chronic stress signals enter the TME

**Fig. 3.**
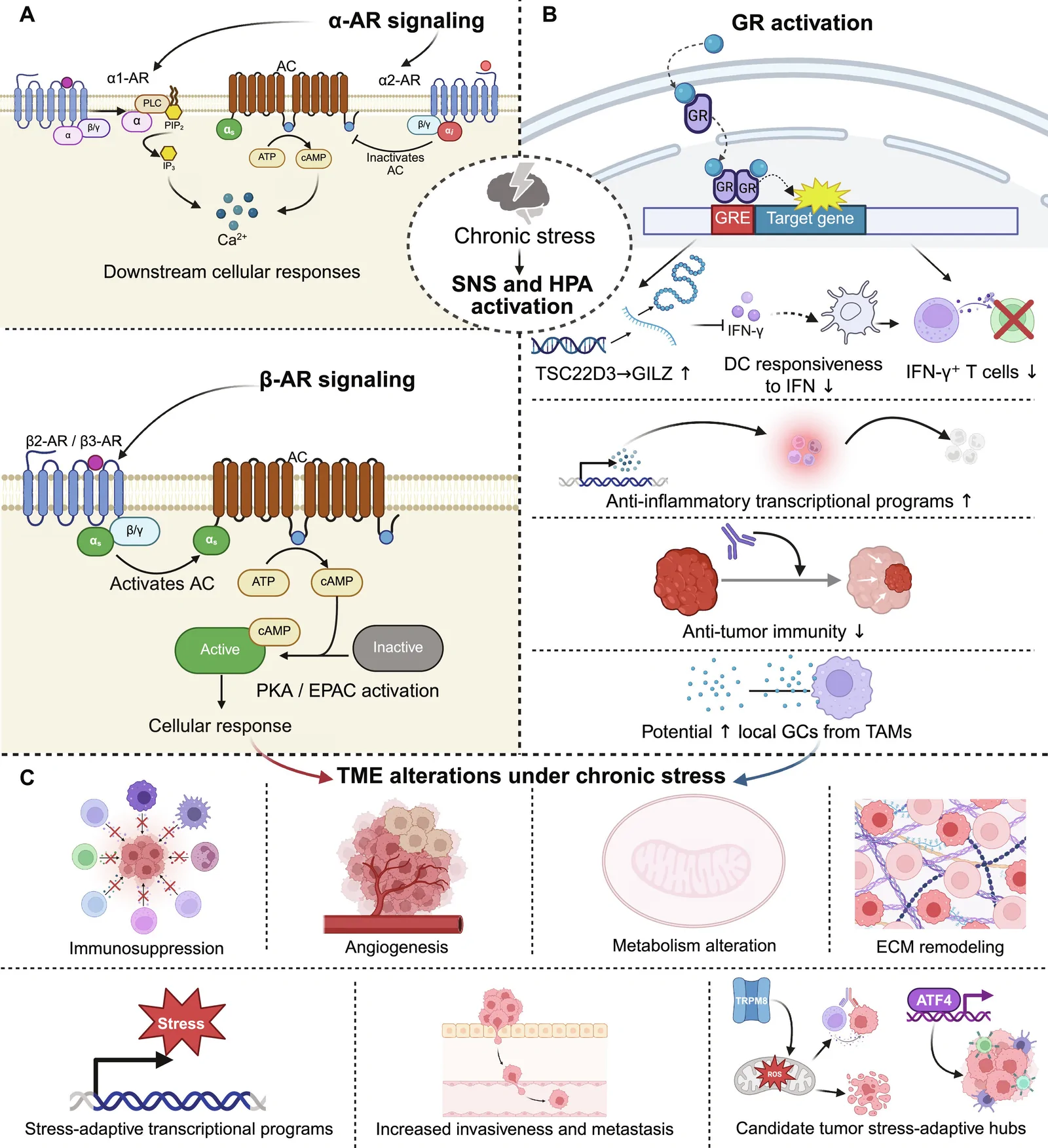
Mechanisms underlying tumor microenvironment remodeling under chronic stress. This schematic summarizes how chronic stress reshapes the TME through sustained activation of the SNS and the HPA axis, thereby transmitting neuroendocrine signals to tumor, immune, vascular, and stromal compartments. (A) Cellular adrenergic signaling is depicted: α₁-AR activates the PLC–IP₃–Ca^2+^ pathway, α₂-AR inhibits AC, and β-ARs activate AC to increase cAMP, leading to PKA/EPAC-dependent responses. (B) Ligand-bound GR translocates to the nucleus and regulates GRE-driven transcription, inducing anti-inflammatory and prosurvival programs, including TSC22D3/GILZ-associated responses, with downstream immunological consequences such as impaired dendritic-cell responsiveness to interferons, reduced IFN-γ^+^ T cell and NK cell effector functions, and expansion or functional skewing of immunosuppressive populations such as TAMs and MDSCs. (C) These coordinated alterations culminate in hallmark TME changes under chronic stress: immunosuppression, enhanced angiogenesis, metabolic reprogramming, ECM remodeling, stress-adaptive tumor-cell programs, and increased invasiveness and metastasis. The lower-right candidate module highlights emerging noncanonical tumor-cell stress-adaptive hubs beyond classical SNS/HPA signaling. TRPM8/ion-channel-associated redox stress may increase mitochondrial ROS and ULBP1-dependent NKG2D–NK recognition, whereas ISR–ATF4–LCN2 signaling has been linked to T cell exclusion and immunosuppressive macrophage infiltration. These pathways are presented as candidate stress-adaptive nodes that require validation in tumor-bearing chronic stress models, rather than as established direct outputs of psychological stress. This figure was generated with BioRender (https://biorender.com). AC, adenylyl cyclase; ATF4, activating transcription factor 4; cAMP, cyclic adenosine monophosphate; ECM, extracellular matrix; EMT, epithelial–mesenchymal transition; EPAC, exchange protein directly activated by cAMP; GR, glucocorticoid receptor; GRE, glucocorticoid response element; HPA, hypothalamic pituitary adrenal axis; IFN-γ, interferon γ; IL-6, interleukin-6; IL-8, interleukin-8; ISR, integrated stress response; LCN2, lipocalin 2; MDSCs, myeloid-derived suppressor cells; MMPs, matrix metalloproteinases; NKG2D, natural killer group 2 member D; NK, natural killer; PGE₂, prostaglandin E2; PKA, protein kinase A; PLC, phospholipase C; ROS, reactive oxygen species; SNS, sympathetic nervous system; TAMs, tumor-associated macrophages; TME, tumor microenvironment; TRPM8, transient receptor potential melastatin 8; *TSC22D3*/GILZ, TSC22 domain family protein 3 (glucocorticoid-induced leucine zipper); ULBP1, UL16-binding protein 1; VEGF, vascular endothelial growth factor; VEGF-C, vascular endothelial growth factor C; α1-AR, α1-adrenergic receptor; α2-AR, α2-adrenergic receptor; β-AR, β-adrenergic receptor; αs, stimulatory G protein α subunit.

Chronic stress reshapes the TME primarily through sustained adrenergic and GC signaling. Sympathetic activation increases intratumoral catecholamines originating from the circulation and local sympathetic fibers [17]. In parallel, HPA activation elevates circulating GCs, which readily enter TME-resident cells [173]. Tumor hypoxia can further amplify both β-adrenergic and GC tone within tumors [174]. At the receptor level, α-adrenergic receptors (α1 and α2) belong to the G protein-coupled receptor (GPCR) superfamily, a major class of therapeutic targets [175]. Specifically, α1-AR activates PLC–IP₃/DAG signaling to increase cytosolic Ca^2+^, whereas α2-AR inhibits adenylyl cyclase (AC), thereby lowering cAMP levels and downstream Ca^2+^ signaling (Fig. 3) [16,176,177]. β-Adrenergic receptors are widely expressed across tumor, stromal, and immune compartments, with β2-AR and β3-AR often serving as dominant mediators in cancer [178,179]. Catecholamine binding activates AC, elevates cAMP, and engages PKA and exchange protein directly activated by cAMP (EPAC), reprogramming transcriptional networks controlling inflammation, angiogenesis, invasion, and metastasis [87,180,181]. GCs derive from adrenal production and, in some settings, local synthesis within the TME [182]. Ligand-activated GR translocates to the nucleus and regulates gene transcription [183]; notably, GR-driven induction of TSC22 domain family protein 3 (TSC22D3) can dampen dendritic-cell type-I interferon responses and limit activation of interferon-γ-positive (IFN-γ^+^) T cells (Fig. 3) [184].

In addition to endocrine catecholamine and GC inputs, stress-associated neural remodeling may influence the TME through neurotrophin-dependent tumor–stroma–nerve crosstalk [118,185]. NGF/Trk signaling and related neurotrophin pathways can support tumor innervation, perineural niche formation, invasive behavior, and context-dependent immune/TME remodeling, with particularly relevant evidence emerging from prostate and BC models [119,121,124,126]. These endocrine inputs do not act on a single TME compartment. Rather, they coordinately remodel immune-cell priming and effector function, reshape cytokine networks, and promote vascular and matrix programs that support invasion and dissemination (Fig. 3).

### Immune remodeling in stressed TMEs

Across models, chronic stress lowers the abundance and activity of antitumor immune populations while expanding exhausted and immunosuppressive subsets in the TME [16]. This immune shift can be traced to impaired antigen presentation, altered T cell metabolism and checkpoint programs, reduced NK cytotoxicity, and myeloid expansion and polarization. Across experimental and clinical models, chronic stress consistently reshapes the immune landscape of the TME, as summarized in Table 1.

**Table 1.** Chronic stress-induced neuroendocrine signaling and immune cell remodeling in the TME

Immune cell types	Neuroendocrine source	Major mediators	Receptor/signaling axis	Key molecular and phenotypic alterations	Metabolic features	Functional consequences in the TME	References
CD8^+^ T cells	SNS	Norepinephrine	β2-AR → cAMP–PKA	Exhaustion ↑ (PD-1, TIM-3); T-bet/IFN-γ ↓	Shift away from glycolysis; impaired mitochondrial function	Functional exhaustion; reduced cytotoxicity	[29,186,189,195,198]
CD4^+^ Th1 cells	SNS and HPA axis	Norepinephrine, glucocorticoids	β-AR/GR	Th1 polarization ↓; IFN-γ ↓	Reduced anabolic metabolism	Weakened support for cytotoxic immunity	[201–204]
Tregs	HPA axis	Glucocorticoids	GR	FOXP3 stabilized; IL-10 ↑	Preferential FAO/OXPHOS reliance	Reinforced immune tolerance	[135,205–207]
NK cells	SNS	Norepinephrine	β2-AR	Perforin/granzyme ↓; KIR balance altered; IFN-γ ↓	Reduced cytotoxic activity and impaired effector function	Impaired tumor-cell killing	[26,211–214]
DCs	HPA axis	Glucocorticoids	GR → GRE-dependent transcription	Maturation/antigen presentation ↓; IL-12/IL-23 ↓; CCR7 migration ↓	Reduced oxidative metabolic capacity	Defective T cell priming; weakened Th1/CTL responses	[186,191,192]
TAMs	SNS and HPA axis	Norepinephrine, glucocorticoids	β-AR/GR	M2-like shift; ARG1/CD206/IL-10/TGF-β ↑	FAO-biased metabolism with enhanced OXPHOS	Immunosuppression with angiogenic/ECM remodeling	[32,33,82,220,221,224,225]
MDSCs	SNS	Norepinephrine	β-AR	Recruitment ↑; ARG1/ROS/COX-2/PGE-2/↑	FAO-favoring metabolic program	Suppressed T cell proliferation and effector function	[33,220,221,227]

ARG1, arginase 1; β-AR, β-adrenergic receptor; β2-AR, β2-adrenergic receptor; cAMP, cyclic adenosine monophosphate; CCR7, C-C motif chemokine receptor 7; CD4, cluster of differentiation 4; CD8, cluster of differentiation 8; CD206, cluster of differentiation 206; COX-2, cyclooxygenase-2; CTL, cytotoxic T lymphocyte; DCs, dendritic cells; ECM, extracellular matrix; FAO, fatty acid oxidation; FOXP3, forkhead box P3; GR, glucocorticoid receptor; GRE, glucocorticoid response element; HPA, hypothalamic pituitary adrenal axis; IFN-γ, interferon γ; IL-10, interleukin-10; IL-12, interleukin-12; IL-23, interleukin-23; KIR, killer-cell immunoglobulin-like receptor; MDSCs, myeloid-derived suppressor cells; NK, natural killer; OXPHOS, oxidative phosphorylation; PD-1, programmed cell death protein 1; PGE₂, prostaglandin E₂; PKA, protein kinase A; ROS, reactive oxygen species; SNS, sympathetic nervous system; TAMs, tumor-associated macrophages; T-bet, T-box transcription factor TBX21; TGF-β, transforming growth factor β; Th1, type 1 helper T cell; TIM-3, T cell immunoglobulin and mucin-domain containing-3; TME, tumor microenvironment; Tregs, regulatory T cells

Melanoma provides a representative setting in which chronic stress intersects with neuroimmune regulation and immune plasticity. In melanoma vaccination models, social disruption stress suppresses tumor protection by impairing DC maturation and migration to draining lymph nodes, thereby reducing tumor-specific IFN-γ-producing CD8^+^ effectors and cytotoxic T lymphocyte (CTL)-mediated killing [186]. Complementary adrenergic stress studies show that β-adrenergic signaling can limit CD8^+^ T cell metabolic reprogramming, including GLUT1-dependent glucose uptake, glycolysis, and mitochondrial fitness, and promote exhausted phenotypes [187]; β-blockade can restore T cell function and improve checkpoint responses in melanoma-relevant settings [188,189]. Together, these findings position melanoma as a stress-sensitive model in which neuroendocrine inputs remodel antigen presentation, T cell metabolism, exhaustion, and effector immunity, linking stress biology to immune plasticity and therapeutic responsiveness.

#### Dendritic cells

**Fig. 4.**
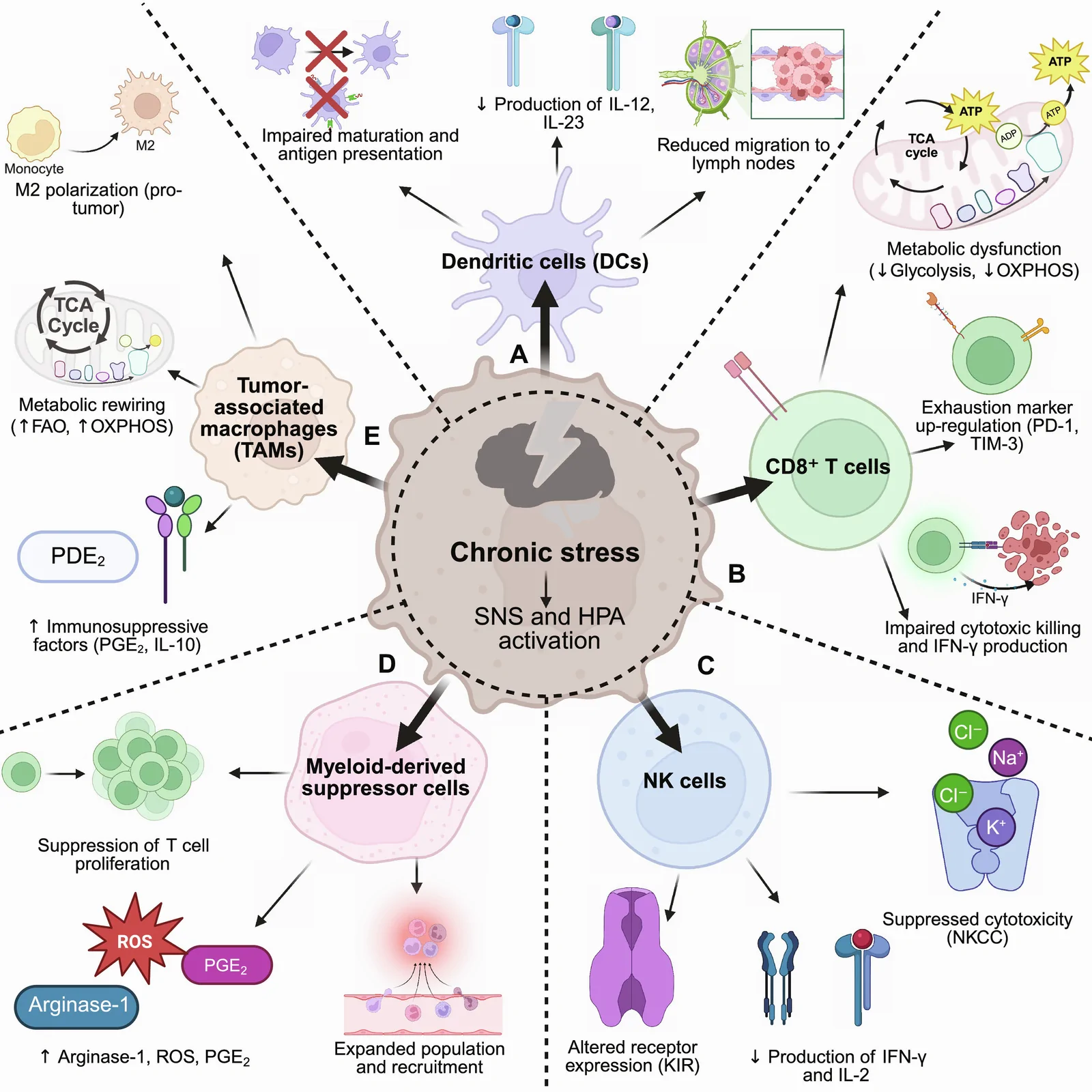
Chronic stress remodels immune-cell function within the tumor microenvironment. Under chronic stress, the following immune-cell changes occur: (A) Dendritic cells show impaired maturation, reduced IL-12 and IL-23 production, and defective migration to draining lymph nodes, resulting in suboptimal T cell priming. (B) CD8^+^ T cells exhibit reduced glycolysis and mitochondrial respiratory capacity, increased expression of exhaustion markers such as PD-1 and TIM-3, and diminished cytotoxicity and IFN-γ production. (C) NK cells show reduced cytotoxic activity and altered activating and inhibitory receptor signaling. (D) MDSCs expand and accumulate in the TME, where they suppress T cell proliferation through ARG1, ROS, and PGE₂-dependent mechanisms. (E) TAMs acquire an M2-like, tumor-promoting phenotype characterized by increased FAO, OXPHOS, IL-10, and PGE₂ production. This figure was generated with BioRender (https://biorender.com). ARG1, arginase 1; SNS, sympathetic nervous system; HPA, hypothalamic pituitary adrenal axis; DCs, dendritic cells; IL, interleukin; CD8^+^ T cells, cluster of differentiation 8-positive T cells; NK cells, natural killer cells; TAMs, tumor-associated macrophages; TME, tumor microenvironment; MDSCs, myeloid-derived suppressor cells; OXPHOS, oxidative phosphorylation; PD-1, programmed cell death protein 1; TIM-3, T cell immunoglobulin and mucin-domain containing-3; IFN-γ, interferon-γ; NKCC, natural killer cell cytotoxicity; KIR, killer-cell immunoglobulin-like receptor; FAO, fatty acid oxidation; PGE₂, prostaglandin E_2_; ROS, reactive oxygen species; ATP, adenosine triphosphate; ADP, adenosine diphosphate; TCA cycle, tricarboxylic acid cycle; Cyt C, cytochrome c; α-KG, α-ketoglutarate; CoA, coenzyme A; PDE2, phosphodiesterase 2.

DCs are essential for tumor antigen presentation and initiation of adaptive antitumor immunity [190]. In melanoma models, chronic stress or GC exposure prevents immature DCs from completing maturation and from efficiently priming Th1 (type 1 helper T cell) and CD8^+^ T cells, whereas the function of already mature DCs is largely preserved [186,191]. Under stress, DC maturation markers [CD40, CD68, major histocompatibility complex class II (MHC II)] decline and antigen presentation together with IL-6, IL-12, and IL-23 production is reduced [192]. Stress also impairs DC migration to draining lymph nodes, directly weakening in vivo priming of CD8^+^ T cells (Fig. 4A) [186]. Mechanistically, the GC-inducible factor TSC22D3 interacts with Ras, nuclear factor κB (NF-κB), and CCAAT/enhancer-binding protein (C/EBP) to suppress type I interferon responses and IFN-γ^+^ T cell activation within DCs [184]. In melanoma, these defects hinder cytotoxic T cell maturation, reflected by reduced killer cell lectin-like receptor subfamily G member 1 (KLRG-1) expression [186]. Accordingly, poly(lactic-co-glycolic acid) microsphere (PLGA-MS) vaccination can expand CD8^+^ T cells and provide prophylactic/therapeutic protection, but chronic stress reduces its protective effect, indicating that DC suppression must be considered in therapeutic settings [186,193].

#### T lymphocytes

T lymphocytes are the principal effectors of adaptive antitumor immunity, and under chronic stress, supportive subsets decline in number or function, while immunosuppressive populations expand [194]. When DC maturation is impaired, CTLs decrease in both healthy and tumor-bearing mice [186,195]. Endogenous GCs blunt DC responsiveness to type I interferons and T cell responses to IFN-γ, thereby limiting differentiation and activation in the TME [184]. GC signaling also promotes dysfunctional CD8^+^ phenotypes with elevated programmed cell death protein 1 (PD-1), T cell immunoglobulin and mucin-domain containing-3 (TIM-3), and lymphocyte activation gene-3 (LAG-3) (Fig. 4B) [182]. In parallel, β-adrenergic input suppresses T cell receptor (TCR) signaling in melanoma and colon cancer models [189].

Beyond signaling, chronic stress alters the metabolic program of T cells. Normally, TCR engagement increases glycolysis and oxidative phosphorylation to meet biosynthetic demands [196]. Adrenergic signaling reduces GLUT1 in CD8^+^ T cells, lowering glucose uptake and glycolysis, and inhibits mitochondrial respiration during activation [187]. β-Blockade can reverse these effects in tumor-infiltrating lymphocytes (TILs), increasing CD28 and improving IFN-γ production, proliferation of antigen-specific CD8^+^ cells, and cytolytic capacity (Fig. 4B) [29,186]. Stress-induced β-adrenergic signaling increases the expression of immune checkpoints (PD-1, TIM-3, and LAG-3), whereas β-blockade lowers multiple checkpoint transcripts in CD8^+^ TILs, including carcinoembryonic antigen-related cell adhesion molecule 1 (CEACAM1), CD160, B and T lymphocyte attenuator (BTLA), programmed death-ligand 1 (PD-L1), and T cell immunoreceptor with Ig and ITIM domains (TIGIT) [189].

These stress pathways intersect with immunotherapy, particularly with antibodies targeting the immune checkpoint molecules PD-1 and CTLA-4, as well as the costimulatory receptor tumor necrosis factor receptor superfamily member 9 (4-1BB) [29,186,195,197]. Conversely, positive environmental stimulation (eustress) can partially reverse this by strengthening CD8^+^ antitumor immunity and overcoming resistance to blockade of PD-1/L1 [198]. In non-small cell lung cancer (NSCLC), corticosteroid use correlates with poorer outcomes and reduced checkpoint-inhibitor efficacy, although mechanisms remain unclear [199,200].

Help from CD4^+^ T cells remains critical for effective cytotoxic responses, in part via CD40 ligand (CD40L)–CD40 licensing of DCs, and the cytokine milieu guides CD4^+^ differentiation [201,202]. β2-Adrenergic activation suppresses Th1 cytokines and proliferation; naïve and Th1 cells express β2-AR, whereas Th2 cells do not, biasing differentiation toward Th2 under adrenergic tone [203,204]. Tregs, typically characterized by a CD4^+^, CD25^+^, and forkhead box P3-positive (FOXP3^+^) phenotype, secrete interleukin-35 (IL-35), IL-10, and transforming growth factor-β (TGF-β) and generally promote tumor growth; the GC-induced protein glucocorticoid-induced leucine zipper (GILZ) enhances Treg development via TGF-β/SMAD signaling and FOXP3 [205,206]. The Th17/Treg balance is important in cancer, but how chronic stress reshapes this axis during tumor progression remains undefined [202]. In ultraviolet (UV)-induced squamous cell carcinoma, stressed mice show increased suppressive CD25^+^ cells, consistent with expansion of immunosuppressive T cell programs under stress [135,207].

#### NK cells

NK cells are frontline innate effectors against cancer, commonly assessed by NKCC [208,209]. Surgical stress lowers NKCC and reduces NK cell Fas ligand and CD11a in melanoma or Lewis lung carcinoma-bearing mice [89]. Similar declines occur in stressed rodent models of leukemia and BC [210]. In patients, psychological distress correlates with impaired NK function: Women with epithelial ovarian cancer show reduced NK cytotoxicity in TILs, and highly stressed BC patients exhibit impaired NK lysis linked to altered killer immunoglobulin-like receptor (KIR) expression (Fig. 4C) [211,212]. Mechanistically, perioperative NK suppression is driven largely by catecholamine surges [89]. Epinephrine and norepinephrine blunt NK cytotoxicity and reduce IL-2, IFN-γ, and IL-12 production through receptor-dependent programs requiring transcription and protein synthesis (Fig. 4C) [213,214]. GCs can also inhibit NKCC but generally act as secondary mediators compared with catecholamines [26].

Beyond this neuroendocrine suppression of NK cells, emerging tumor-cell-centered evidence indicates that noncanonical stress-adaptive programs can determine not only tumor-cell survival but also immune visibility to NK cells. In metastatic melanoma, pharmacological modulation of the TRPM8 ion channel induces mitochondrial ROS-dependent apoptotic priming and increases ULBP1 expression, thereby enhancing NK cell-mediated cytotoxicity through ULBP1/NKG2D engagement [42]. This finding suggests that membrane-associated stress-sensing pathways may generate an immunogenic vulnerability when redox stress exceeds adaptive buffering capacity, linking tumor redox imbalance, mitochondrial fitness, and innate immune surveillance.

#### Tumor-associated macrophages and MDSCs

Tumor-associated macrophages (TAMs) are often the dominant myeloid infiltrate in the TME. Depression and psychosocial stress increase intratumoral TAMs and are associated with poor prognosis and therapy resistance, consistent with tumor-promoting myeloid roles [215,216]. Catecholaminergic signaling also expands MDSCs (CD11b^+^Gr1^+^) [217]. Recruitment can be gated by chemokine programs: Chronic stress increases tumor-cell C-X-C motif chemokine ligand 2 (CXCL2) and CXCL3 and induces C-X-C motif chemokine receptor 2 (CXCR2) on myeloid cells, enabling recruitment via the CXCL2/CXCL3–CXCR2 axis [218]. Adrenergic input can also elevate tumor C-C motif chemokine ligand 2 (CCL2), increasing CD14^+^/CD68^+^ macrophage influx [219]. In parallel, GC–GR signaling down-regulates low-density lipoprotein receptor-related protein 1 (LRP1) and up-regulates signal regulatory protein α (SIRPα) in macrophages, shifting the LRP1/SIRPα balance to inhibit tumor-cell phagocytosis [219].

Chronic stress further reprograms myeloid-cell metabolism toward immune suppression. In MDSCs and TAMs, β₂-adrenergic signaling decreases glycolysis while increasing oxidative phosphorylation and fatty acid oxidation (FAO) (Fig. 4D and E) [33,220,221]. FAO, in turn, elevates COX-2 and prostaglandin E₂ (PGE₂) [33,221]; PGE₂ can recruit neutrophils and macrophages and suppress CD8^+^ T cell proliferation and IFN-γ production [222]. Consistently, reward-system activation reduces bone marrow norepinephrine, decreases MDSC output, and weakens MDSC-mediated suppression in tumor-bearing mice [223]. Catecholamines also bias macrophage polarization toward an M2-like state, supporting metastasis and angiogenesis through prometastatic gene programs, proangiogenic mediators, and perivascular matrix degradation (Fig. 4E) [32,224]. In response to β-adrenergic cues, TAMs can signal via COX-2/PGE₂ to induce vascular endothelial growth factor-C (VEGF-C), a driver of lymphatic remodeling that promotes lymphangiogenesis, lymphatic dissemination, and nodal metastasis [82,225].

Together, these pathways illustrate how chronic stress reshapes innate immunity to favor tumor progression. Stress can directly weaken TIL and NK function through epigenetic and metabolic programming and indirectly disable immune support from macrophages and DCs, undermining immunotherapy and permitting unchecked tumor growth [226]. MDSCs represent a central suppressive population in both cancer and chronic stress. Surgical stress increases their numbers in the TME, and other stress models report concurrent rises in MDSCs and Tregs [227]. TAM expansion is consistently observed in both clinical and preclinical settings. For instance, in patients with prostate cancer, higher depression scores correlate with greater CD68^+^ TAM density. Similarly, in ovarian cancer models, daily restraint stress increases the infiltration of CD68^+^ macrophages [208,216,228]. Beyond myeloid cells, CAFs remodel the microenvironment through cell contact, growth-factor release, and matrix regulation; α₂-adrenergic activation accelerates fibroblast growth and raises growth-factor levels in the TME [229–231]. Collectively, chronic stress reinforces immunosuppressive myeloid–stromal circuits while weakening cytotoxic effector arms, thereby coupling immune escape to cytokine, vascular, and matrix programs that support invasion and dissemination.

### Cytokine programs in stressed TMEs

**Fig. 5.**
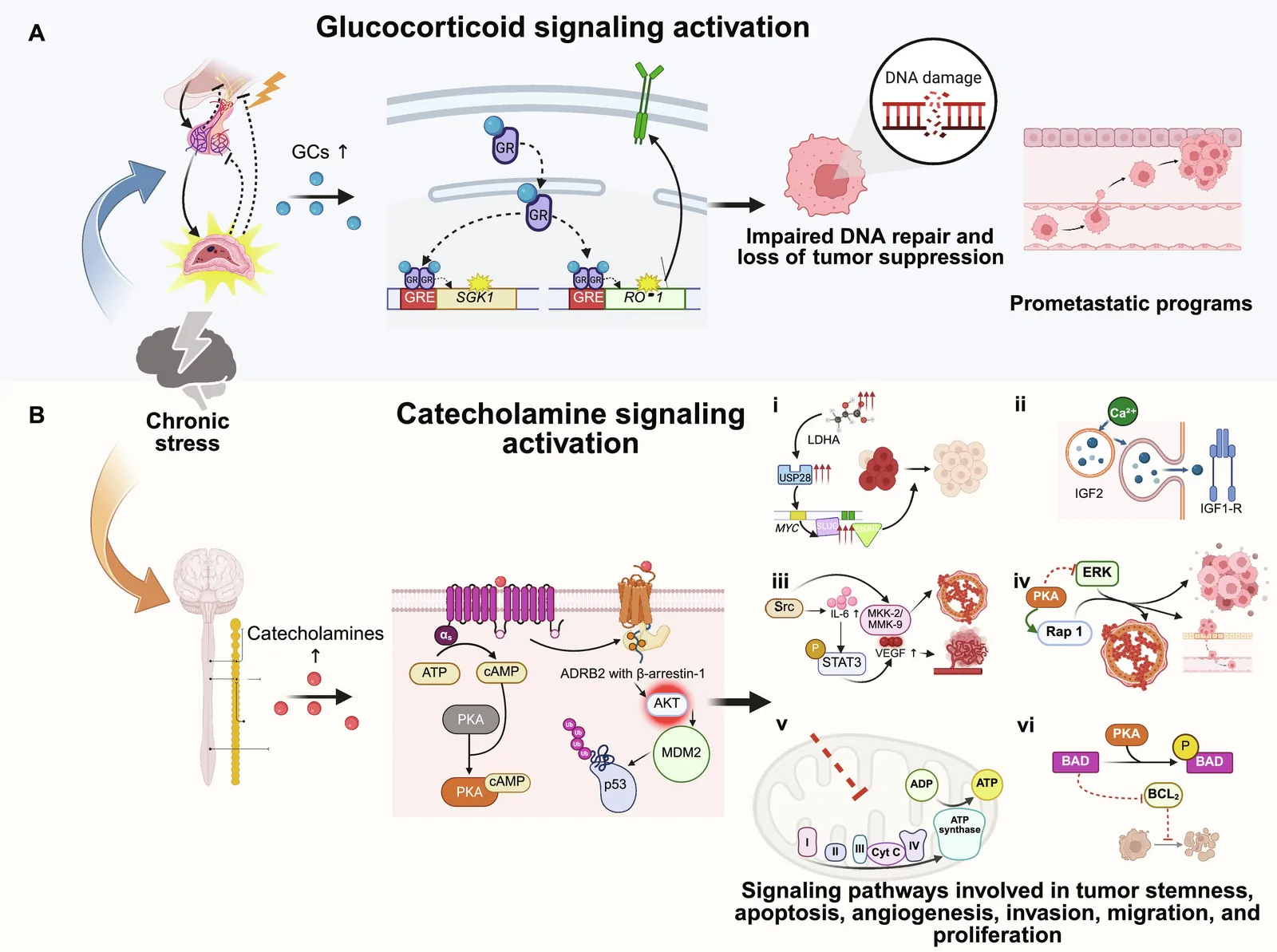
Chronic stress activates glucocorticoid and catecholamine signaling to promote tumor survival, plasticity, and progression. Chronic stress sustains activation of the HPA axis and SNS, resulting in elevated GCs and catecholamines (norepinephrine/epinephrine). (A) Glucocorticoid signaling activation: Increased GCs engage the GR, which translocates to the nucleus and binds GREs to induce stress-responsive transcriptional programs (illustrated by SGK1 and ROR1 up-regulation). These GR-driven outputs are linked to impaired genome maintenance (e.g., reduced DNA repair capacity) and loss of tumor-suppressive control, thereby facilitating tumor initiation and progression. (B) Catecholamine signaling activation: SNS-derived catecholamines activate β-adrenergic signaling (illustrated by ADRB2), stimulating adenylyl cyclase–cAMP–PKA pathways and β-arrestin–dependent signaling. Downstream effects include AKT/MDM2–p53 modulation and multiple protumor programs such as (i) stemness-promoting LDHA activation and USP28–MYC–SLUG signaling, (ii) Ca^2+^-dependent IGF2 release and IGF-1R activation, (iii) Src-driven inflammatory/angiogenic signaling (e.g., IL-6/STAT3, VEGF, and MMP-2/MMP-9) that supports invasion and neovascular remodeling, (iv) Rap1 activation with ERK-pathway rewiring to enhance migration and proliferation, (v) suppression of oxidative phosphorylation that contributes to metabolic reprogramming, and (vi) BAD phosphorylation with inhibition of apoptosis. Together, the figure summarizes how coordinated GC/GR and adrenergic/PKA signaling provides a mechanistic bridge from chronic stress to tumor aggressiveness and therapy-relevant resistance phenotypes. This figure was generated with BioRender (https://biorender.com). ADRB2, adrenergic receptor β2; AKT, AKT serine/threonine kinase; BAD, BCL2-associated agonist of cell death; cAMP, cyclic adenosine monophosphate; ERK, extracellular signal-regulated kinase; GCs, glucocorticoids; GR, glucocorticoid receptor; GREs, glucocorticoid response elements; HPA, hypothalamic pituitary adrenal axis; IGF-1R, insulin-like growth factor 1 receptor; IGF2, insulin-like growth factor 2; IL-6, interleukin-6; LDHA, lactate dehydrogenase A; MDM2, mouse double minute 2; MMP-2/MMP-9, matrix metalloproteinase 2/9; MYC, MYC proto-oncogene; PKA, protein kinase A; ROR1, receptor tyrosine kinase-like orphan receptor 1; SGK1, serum/glucocorticoid-regulated kinase 1; SLUG, Snail family transcriptional repressor 2 (SNAI2); SNS, sympathetic nervous system; STAT3, signal transducer and activator of transcription 3; USP28, ubiquitin-specific peptidase 28; VEGF, vascular endothelial growth factor; α_s_, stimulatory G protein α subunit.

Chronic stress sustains activation of the HPA axis and SNS, leading to elevated GCs and catecholamines (norepinephrine/epinephrine) [226]. GCs engage the GR, which translocates to the nucleus and binds GC response elements (GREs), inducing transcriptional programs such as SGK1 and ROR1 up-regulation [232–234]. These GR-mediated outputs impair genome maintenance and DNA repair while reducing tumor-suppressive control, thereby facilitating tumor initiation and progression [226,235]. Concurrently, catecholamines activate β-adrenergic receptors, especially the β2-AR, thereby triggering AC–cAMP–PKA and β-arrestin-dependent signaling pathways (Fig. 5) [180]. Downstream signaling modulates AKT/MDM2–p53, inhibits apoptosis via BAD phosphorylation, and promotes tumor-supportive programs including stemness, Ca^2+^-dependent growth-factor release, inflammatory/angiogenic signaling, migration/proliferation via ERK/Rap1, and metabolic reprogramming (Fig. 5) [7,180,236]. Together, sustained GC/GR and adrenergic signaling provide a mechanistic bridge linking chronic stress to tumor aggressiveness and therapy resistance.

Within the TME, these neuroendocrine signals drive a profound shift in cytokine profiles toward programs that support tumor growth, invasion, and immune evasion. In melanoma, GCs reduce IFN-γ-producing cells and intratumoral IFN-γ [186,191]. In BC models, expanded MDSCs elevate TGF-β1, VEGF, and IL-10 within the TME [237]. GCs further enhance TGF-β signaling by up-regulating TGF-β receptor II on ovarian cancer cells [238]. β-Adrenergic signaling adds a second amplifying layer, increasing neuropeptide Y with TAM trafficking in prostate cancer and elevating IL-6 via TAM activation and tumor-cell secretion [238,239]. In epithelial ovarian cancer patients under chronic stress, TAMs exhibit increased matrix metalloproteinase-9 (MMP-9) [240]. Across multiple models, stress or catecholamine exposure elevates VEGF, MMP-2, and MMP-9 in ovarian and lung cancers; norepinephrine similarly up-regulates VEGF, IL-8, and IL-6 in human melanoma lines [87,241–243]. Epinephrine also boosts PGE₂ in ex vivo human breast and colon explants and in mammary tumors from stressed mice [220]. Broad cytokine profiling in stressed ovarian cancer models reveals increases in platelet-derived growth factor-AA (PDGF-AA), ENA-78, angiogenin, VEGF, granulocyte-macrophage colony-stimulating factor (GM-CSF), IL-5, lipocalin-2, macrophage migration inhibitory factor (MIF), and transferrin receptor [228]. This stress-driven cytokine milieu feeds directly into vascular remodeling and tissue-remodeling programs, most prominently angiogenesis and lymphangiogenesis, thereby coupling immune suppression with structural pathways that facilitate tumor expansion and dissemination.

### Angiogenesis and vascular remodeling

Chronic stress shifts the TME toward angiogenesis, with increases in VEGF together with IL-6, TGF-β, and MMPs that collectively promote vessel growth in solid tumors [244]. This proangiogenic pattern is observed in stressed rodent models of ovarian, oral, and lung cancers [87,241,243]. In LC, stress also increases endothelial VEGFR-2, amplifying proangiogenic signaling [241]. In BC, stress augments VEGF/fibroblast growth factor 2 (FGF2)-driven angiogenesis by suppressing PPAR-γ [245]. In prostate cancer, β-adrenergic input promotes angiogenesis through histone deacetylase 2 (HDAC2)-mediated repression of thrombospondin-1 [246]. The resulting vasculature is often disorganized and poorly functional, worsening hypoxia and acidosis within the TME [247,248].

### EMT and stromal/matrix remodeling

#### EMT

Chronic stress increases TGF-β1 within the TME; because TGF-β signaling is a core EMT driver, this shift promotes EMT programs and metastatic competence [249]. In stressed BC models, high TGF-β1 induces EMT and accelerates metastasis [237]. In vitro, norepinephrine induces EMT by reducing E-cadherin expression and increasing vimentin expression through β-adrenergic/TGF-β1 pathways involving p-Smad3/Snail or HIF-1α/Snail signaling in gastric, colon, and lung cancer cells [231,250]. Consistently, chronic stress decreases E-cadherin and increases vimentin through the miR-337-3p/STAT3 axis in stressed BC models [251]. Adrenergic receptors also couple to PKA and protein kinase C (PKC), linking stress cues to EMT-related signaling, although direct evidence from stress-hormone models that isolate PKA/PKC-dependent EMT remains limited, and their precise contributions require further study [176,177,180,252–260]. Chronic stress is also associated with gut-microbiota dysbiosis, which can facilitate EMT through microbiota–host interactions [261,262].

#### ECM and related stromal components

Across malignancies, stressed TMEs commonly exhibit increased MMP activity, which drives ECM degradation and thereby facilitates invasion and metastasis [87,240,241,243,263]. GC exposure can further remodel ECM programs; in a rodent Wilms’ tumor model, GCs suppress tenascin-C synthesis even in the presence of local growth-factor stimulation [264]. Beyond matrix enzymes, CAFs remodel the microenvironment through cell contact, growth-factor release, and matrix regulation; α₂-adrenergic activation accelerates fibroblast growth and increases growth-factor levels in the TME [229–231].

Taken together, chronic stress reshapes the TME through sustained adrenergic and GC signaling that (a) impairs antigen presentation and effector immunity while expanding immunosuppressive cell populations, (b) establishes protumor cytokine programs, and (c) promotes vascular and tissue remodeling, including angiogenesis, lymphangiogenesis, EMT, and ECM degradation, which facilitate invasion and dissemination. These interconnected changes provide a coherent framework for understanding how chronic stress shifts the TME toward immune evasion and metastatic progression and how stress biology may influence responses to immunotherapy.

#### Integration of systemic and local stress signaling in tumor progression

Chronic stress promotes tumor progression not only through direct receptor-mediated effects within the TME but also through broader systemic metabolic and inflammatory conditioning that creates a permissive host context for malignancy. Sustained activation of the SNS and the HPA axis reshapes glucose, lipid, and amino acid metabolism, alters nutrient availability and bioenergetic states, and disturbs immune and inflammatory homeostasis across peripheral tissues. These systemic changes can support tumor-cell survival, anabolic growth, and adaptation to cytotoxic stress, thereby providing a metabolic backdrop that favors disease progression [16,226].

At the local level, catecholamines and GCs act through adrenergic receptors and the GR on tumor cells and stromal, vascular, and immune components of the TME, converting this permissive systemic state into concrete protumor outputs. As discussed above, chronic stress suppresses antigen presentation and effector immunity, expands immunosuppressive cell populations, reinforces protumor cytokine signaling, and promotes angiogenesis, EMT, and ECM remodeling [87,229–231,240,241,243,249–251,263]. These systemic and local pathways therefore act in concert rather than in isolation: Systemic metabolic reprogramming and inflammatory disequilibrium increase tumor permissiveness, whereas local receptor-mediated signaling drives immune evasion, invasion, dissemination, and reduced therapeutic responsiveness within the TME. Viewed together, this integrated framework helps explain how chronic stress simultaneously promotes tumor growth, metastasis, and treatment resistance, and it supports the need for therapeutic strategies that address both systemic stress biology and local tumor-associated signaling networks [16,226].

## Cancer-Specific Mechanisms Linking Chronic Stress to Tumor Progression

Across cancer types, chronic stress converges on a shared catecholamine–adrenergic backbone, most consistently involving β2-AR signaling, but the dominant tumor-promoting output differs by tissue context. Metastasis and niche conditioning are most prominent in breast and lung cancer, autophagy-linked survival in gastric cancer, immune skewing in skin and colon cancer, anti-apoptotic and angiogenic signaling in prostate cancer, invasive gene activation in pancreatic cancer, and combined cell-cycle/ECM/immune remodeling in lymphoma. Table 2 compares these cancer-specific patterns by dominant stress-responsive program, mechanistic signature, biological output, translational implication, and evidence maturity.

**Table 2.** Cancer-specific stress-responsive mechanisms and tumor-promoting outputs

Cancer context	Dominant stress-responsive program	Mechanistic signature	Predominant tumor-promoting output	Translational implication	Evidence maturity in this section	Refs.
Breast cancer	β2-AR-centered adrenergic signaling, layered with GR, prostaglandin, cholinergic, and microbiota-linked inputs	β2-AR-driven invadopodia; LDHA–USP28–MYC–SLUG metabolic/stem-like program; CCL2/CCR2 and CXCL2/CXCR2 niche conditioning; Blautia–acetate–CD8^+^ T cell axis	Invasion, stem-like traits, lymphatic and distant metastasis, premetastatic niche formation, and impaired cytotoxic immunity	Nonselective β-blockade may be most relevant; combination strategies could target glycolysis, inflammatory remodeling, or stress-linked immunometabolism	Advanced preclinical evidence with selected clinical correlative support	[7,128,280]
Gastric cancer	Sympathetic nerve activity and norepinephrine–β2-AR signaling	β2-AR–AMPK–ULK1 autophagy axis, with additional NF-κB/AP-1/CREB/STAT3/MAPK-linked survival, invasion, angiogenic, and matrix-remodeling programs	Tumor growth, survival, invasion, angiogenesis, liver/lung colonization, and lymphatic metastasis	β-Adrenergic blockade or sympathetic dampening; autophagy-related markers may support stratification	Strong mechanistic evidence with receptor-expression and clinicopathologic correlations	[97,133]
Lung cancer	Norepinephrine–β-AR–PKA–Ca^2+^ signaling, with stress-conditioned immune and metastatic-niche remodeling	VDCC–IGF2–IGF-1R activation; CCL2/CCR2-dependent macrophage recruitment; tryptophan–kynurenine and T cell remodeling in stress-intervention models	Tumor initiation, growth advantage, immune escape, macrophage-rich lung niche formation, and metastatic colonization	β-Blockers, calcium-channel blockade, or IGF-1R-directed strategies merit context-specific testing; stress-immunomodulatory approaches remain exploratory	Strong for adrenergic–IGF signaling; moderate for immune-intervention mechanisms	[58,63,134,180]
Skin cancer (mainly UV-induced SCC models)	Chronic stress superimposed on UV-induced immune injury	Reduced type 1 cytokine production, weakened protective T cell responses, and expansion of regulatory or suppressor T cell populations	Impaired antitumor surveillance and permissive outgrowth of UV-damaged epithelial cells	Immune-preserving strategies are conceptually relevant, but broader validation beyond UV-induced SCC models is needed	Model-restricted evidence	[7,135]
Prostate cancer	Epinephrine–β2-AR signaling in tumor and endothelial compartments	β2-AR–PKA-mediated BAD phosphorylation; endothelial metabolic remodeling toward a proangiogenic state	Tumor-cell survival and angiogenesis under stress	β-Adrenergic blockade may complement anti-survival or antiangiogenic strategies	Focused mechanistic preclinical evidence	[297,298]
Colon cancer	Stress-induced norepinephrine/corticosterone signaling and immune polarization	Th1-to-Th2 shift with IFN-γ reduction and IL-4/IL-10 induction; TSC22D3 and COX-2/PGE₂–VEGF-C as additional immunovascular programs	Accelerated tumor growth, weakened cell-mediated immunity, invasion, and lymphatic remodeling	Immune rebalancing and combined β-adrenergic/GR/COX-2 axis modulation require further testing	Moderate preclinical evidence	[7,184]
Pancreatic cancer	Catecholamine–β2-AR signaling	β2-AR-dependent activation of invasion-related gene programs, motility, and tissue penetration	Local invasion and distant dissemination	β-Blockade is a plausible adjunctive strategy, but evidence breadth remains limited	Focused but relatively narrow mechanistic evidence	[265,301]
Lymphoma models	Chronic stress effects on tumor-cell cycling, ECM remodeling, and immune function	Cyclin A2/D1/D3 up-regulation; CDK inhibitor suppression; MMP-2/MMP-9 induction; TIMP reduction; immune-cell-dependent tumor promotion	Tumor growth, migration, invasion, spontaneous metastasis, and impaired antitumor immunity	SSRI-associated attenuation and immune restoration are hypothesis-generating, but tumor-cell-intrinsic effects cannot be excluded	Model-dependent preclinical evidence	[302,384]

AMPK, AMP-activated protein kinase; AP-1, activator protein 1; BAD, BCL2-associated agonist of cell death; β-AR, β-adrenergic receptor; β2-AR, β2-adrenergic receptor; CCL2, C-C motif chemokine ligand 2; CCR2, C-C motif chemokine receptor 2; CD8, cluster of differentiation 8; CDK, cyclin-dependent kinase; COX-2, cyclooxygenase-2; CREB, cAMP response element-binding protein; CXCL2, C-X-C motif chemokine ligand 2; CXCR2, C-X-C motif chemokine receptor 2; ECM, extracellular matrix; GR, glucocorticoid receptor; IFN-γ, interferon γ; IGF, insulin-like growth factor; IGF-1R, insulin-like growth factor 1 receptor; IGF2, insulin-like growth factor 2; IL-4, interleukin-4; IL-10, interleukin-10; LDHA, lactate dehydrogenase A; MAPK, mitogen-activated protein kinase; MMP-2, matrix metalloproteinase 2; MMP-9, matrix metalloproteinase 9; MYC, MYC proto-oncogene, bHLH transcription factor; NF-κB, nuclear factor κB; PGE₂, prostaglandin E₂; PKA, protein kinase A; SCC, squamous cell carcinoma; SLUG, Snail family transcriptional repressor 2; SSRI, selective serotonin reuptake inhibitor; STAT3, signal transducer and activator of transcription 3; Th1, type 1 helper T cell; Th2, type 2 helper T cell; TIMPs, tissue inhibitors of metalloproteinases; TSC22D3, TSC22 domain family member 3; ULK1, unc-51-like kinase 1; USP28, ubiquitin-specific peptidase 28; UV, ultraviolet; VDCC, voltage-dependent calcium channel; VEGF-C, vascular endothelial growth factor C

### Breast cancer

In BC, chronic stress promotes tumor progression primarily through adrenergic signaling in malignant and stromal compartments, metabolic reprogramming, stem-like phenotypes, metastatic niche conditioning, and immune suppression. The strongest direct evidence centers on β2-AR signaling. In MDA-MB-231HM BC models, activation of β2-AR signaling promotes invadopodia formation and invasive behavior, whereas silencing β2-AR expression abolishes stress-driven metastasis without necessarily altering primary tumor size [129]. These findings identify tumor-cell β2-AR signaling as a direct driver of stress-enhanced dissemination and indicate that β-adrenergic signaling in both malignant and stromal compartments cooperates to promote metastatic progression [80,82,84,86–88,129,265–268].

The effects of adrenergic blockade appear context-dependent. β1-AR, β2-AR, and β3-AR are present in stromal and malignant cells [74,269–271]. In MDA-MB-231HM cells, β2-AR is the dominant functional subtype, whereas other tumor models express β1-AR and β3-AR [265,268,272–274]. This receptor-subtype heterogeneity may explain why nonselective β-blockers such as propranolol, rather than β1-AR-selective agents, have been more consistently associated with improved BC outcomes in some clinical datasets [59]. α-Adrenergic regulation further complicates this axis: In an orthotopic BC model, nonselective α blockade with phentolamine reduced stress-driven tumor growth and metastasis, but under nonstress conditions increased tumor burden, an effect prevented by propranolol [267]. This suggests that presynaptic α2 autoreceptors restrain catecholamine release and thereby tune downstream β-adrenergic tumor-promoting pathways [267].

Chronic stress also promotes BC stem-like properties through an epinephrine–β2-AR–LDHA–USP28–MYC–SLUG program. Stress-induced epinephrine activates β2-AR, increases LDHA expression and activity, and shifts tumor cells toward glycolytic metabolism [128]. Increased LDHA-driven lactate production lowers local pH and strengthens the USP28–MYC interaction, while epinephrine also elevates USP28, stabilizing MYC and sustaining SLUG transcription [128]. This signaling program supports self-renewal, stem-like traits, abnormal differentiation, and malignant progression [7,128]. Clinically, higher serum epinephrine correlates with activation of the LDHA/USP28/MYC/SLUG axis, and LDHA has been reported as an independent prognostic marker in BC [128]. Preclinical data further suggest that high-dose vitamin C, as a competitive LDHA inhibitor, can disrupt this stress-driven β2-AR–LDHA–SLUG cascade and reduce BC growth [7,128].

Several additional stress-responsive programs converge on invasion, angiogenesis, lymphangiogenesis, and metastatic competence. Psychosocial stress can activate corticotropin-releasing factor (CRF)–focal adhesion kinase (FAK) signaling, GC–GR–ROR1 signaling, and COX-2/PGE₂–VEGF-C remodeling, thereby promoting cytoskeletal reorganization, invasion, angiogenesis, and lymphatic dissemination [7]. These mechanisms are best interpreted as convergent stress-responsive outputs rather than separate isolated pathways: Catecholamine, GC, prostaglandin, and lymphangiogenic programs collectively lower barriers to invasion and distant spread [7].

Chronic stress can also condition distant metastatic niches. In bone, sympathetic signaling acts through β-adrenergic receptors expressed by bone-lineage cells and tumor cells, shifting bone remodeling toward resorption and creating a more permissive niche for BC colonization [67,275–277]. Adrenergic agonists raise osteoblast-derived RANKL, promoting osteoclastogenesis [276], while the CXCL12/SDF1–CXCR4 axis supports homing and survival of BC cells in bone [278–281]. RANKL further enhances the migration of melanoma, breast, prostate, and lung cancer cells and promotes skeletal metastasis in experimental systems [282–284]. Clinically, coexpression of RANK and CXCR4 identifies BC patients at high risk for bone metastasis [280].

In the lung, chronic stress prepares a premetastatic niche through both adrenergic-myeloid and cholinergic-neutrophil programs. β2-AR/CCL2/CCR2 signaling increases CCL2 in pulmonary stromal cells and CCR2 in monocytes and macrophages, recruiting myeloid cells to the lung and facilitating tumor colonization; propranolol disrupts this prometastatic circuit [81]. In parallel, chronic stress induces a cholinergic program in pulmonary epithelial neuroendocrine cells, increasing acetylcholine production, recruiting neutrophils through the CXCL2–CXCR2 axis, and enhancing neutrophil extracellular trap formation, which enables NETotic neutrophils to capture disseminated BC cells [285]. Clinically, lungs from patients with BC metastases show higher choline acetyltransferase expression than those from patients with nonneoplastic pulmonary disease [285].

Stress-associated depression can further accelerate BC progression through the gut microbiota–acetate–CD8^+^ T cell axis. Reductions in Blautia, a dominant acetate-producing genus, drive stress-associated tumor promotion by lowering acetate availability, thereby weakening CD8^+^ T cell activation under stress [286]. In preclinical BC models with depression-like states, supplementation with Blautia or acetate enhances antitumor CD8^+^ T cell responses and limits tumor progression [286]. Mechanistic studies further show that acetate modulates tumor–immune cell interactions, expands the acetyl-coenzyme A (CoA) pool in CD8^+^ T cells, and increases IFN-γ production [286]. Human correlative data show that BC patients with coexisting depression have lower serum acetate and fewer intratumoral CD8^+^ T cells than nondepressed patients, supporting disruption of the Blautia–acetate axis as a stress-linked immunometabolic mechanism in BC [286].

### Gastric cancer

In gastric cancer, chronic stress promotes tumor growth, survival, invasion, and metastasis mainly through sympathetic activation and β2-AR-centered signaling. The strongest mechanistic evidence links norepinephrine–β2-AR activation to autophagy and AMPK–ULK1 signaling. Chronic stress raises norepinephrine and corticosterone in plasma and gastric tissue and accelerates tumor growth in both subcutaneous and orthotopic xenografts [133]. Physiologic norepinephrine delivery through osmotic pumps also enhances tumor growth in mice, supporting norepinephrine as a direct tumor-promoting stress mediator in gastric cancer [133].

Selective β2-AR stimulation induces autophagy in gastric cancer cells, and this autophagic program supports catecholamine-induced proliferation and survival in vitro [133]. Immobilization stress also engages autophagy-related signaling downstream of β2-AR [133]. In clinical gastric tumor samples, β2-AR expression correlates with SQSTM1, a central autophagy adaptor [133]. Mechanistically, norepinephrine activates β2-AR, which signals through the AMPK–ULK1 axis to sustain autophagy, energy supply, and proliferative outgrowth [7,133]. *ADRB2* silencing lowers Beclin1 expression, blunts norepinephrine-induced AMPK and ULK1 activation, reduces autophagic activity, and limits gastric cancer cell survival [133]. High β2-AR protein expression correlates with advanced TNM stage and poorer prognosis, supporting β2-AR as both a mechanistic driver and potential therapeutic target [133].

Peripheral sympathetic input is required for stress-enhanced gastric tumor progression in vivo. In gastric cancer, chronic restraint stress promotes tumor growth and metastasis predominantly through sympathetic β2-adrenergic signaling. Stress-induced catecholamines activate β2-AR and downstream MAPK, NF-κB, AP-1, CREB, and STAT3 signaling, thereby enhancing tumor-cell proliferation, migration, invasion, and survival, accompanied by increased VEGF and MMP expression. These effects are attenuated by sympathetic denervation or β-adrenergic blockade. Clinically, elevated tumoral β2-AR expression is associated with larger tumor size, poor differentiation, advanced clinical stage, and lymph-node metastasis [97].

An additional stress-responsive gastric cancer mechanism is the progression of precancerous lesions. In rats, chronic stress accelerates gastric precancerous lesions by promoting inflammation, stimulating proliferation, suppressing apoptosis, disrupting gastrointestinal homeostasis, and altering gastrokine 2 and ghrelin signaling [287].

### Lung cancer

In LC, the strongest stress-linked mechanism involves a norepinephrine–β-adrenergic receptor–PKA–voltage-dependent calcium channel (VDCC)–insulin-like growth factor 2 (IGF2)–insulin-like growth factor 1 receptor (IGF-1R) pathway that promotes tumor initiation and progression. Under chronic stress, norepinephrine activates β-adrenergic receptors and PKA, which phosphorylates L-type VDCCs and raises cytosolic calcium [134]. This calcium influx triggers exocytosis of IGF2, leading to autocrine and paracrine activation of IGF-1R in lung epithelial cells [134]. IGF-1R activation supports transformation and survival, giving premalignant epithelial cells a growth advantage under carcinogen or oncogene stress. β-Adrenergic receptor antagonists, L-type calcium-channel blockers, and IGF-1R inhibitors reduce IGF2 release, IGF-1R phosphorylation, cellular transformation, and stress-induced lung tumorigenesis [134].

Chronic unpredictable stress raises circulating norepinephrine to physiologic ranges and accelerates lung tumor formation in urethane and *Kras*^G12D/+^ mouse models [134]. Continuous norepinephrine delivery via osmotic pumps increases lung tumor burden in vivo, confirming norepinephrine as a tumor-promoting mediator [134]. Although stress can alter cytokine output, reduce NKCC, and suppress T cell mitogenesis [17], stress-enhanced lung tumor growth in immune-deficient models suggests that immune suppression is not the only mechanism [87,180,288–290]. Instead, stress-linked IGF-1R activation in lung epithelial cells appears to be a pivotal biochemical event in lung carcinogenesis [134,291,292]. Tissue-derived IGF2, rather than circulating IGFs, is proposed to mediate this effect, since blocking IGF2 or IGF-1R prevents norepinephrine-induced IGF-1R phosphorylation and transformation [291–294].

This adrenergic–calcium–IGF pathway may also intersect with tobacco-related carcinogenesis. Norepinephrine and the tobacco-specific carcinogen 4-(methylnitrosamino)-1-(3-pyridyl)-1-butanone (NNK) share β-adrenergic signaling as a mechanistic node, suggesting that chronic stress may cooperate with tobacco exposure in lung tumorigenesis [295]. These findings nominate β-blockers and calcium-channel blockers as pharmacologic candidates for mitigating stress-associated LC risk or progression, particularly in populations exposed to persistent psychological stress [58,63,180]. Beyond tumor initiation, chronic stress can also promote lung immune escape, metastasis, and niche formation. GC-induced TSC22D3 suppresses type I interferon programs in DCs and weakens IFN-γ-producing T cell activity [184]. Chronic stress also prepares the lung as a metastatic niche. Stress increases the efficiency with which circulating tumor cells colonize the lung, and this effect is reduced by chemical sympathectomy with 6-OHDA or β-adrenergic blockade with propranolol [81]. Depletion of monocytes or lung macrophages abolishes tumor seeding, whereas isoproterenol increases macrophage infiltration in lung tissue and raises monocyte numbers in blood, spleen, and bone marrow [81]. Mechanistically, isoproterenol up-regulates CCL2 in pulmonary stromal cells and CCR2 in monocytes and macrophages, thereby recruiting macrophages to the premetastatic lung and facilitating colonization [81].

Fluoxetine has also been reported to counteract chronic stress-enhanced LC growth. In C57BL/6 mice, persistent stress accelerates tumor growth, whereas fluoxetine reverses this phenotype, restores serotonin and tryptophan, reduces kynurenine, increases CD4^+^ and CD8^+^ T cells, reduces CD25^+^FOXP3^+^ Tregs, shifts helper responses toward Th1, and decreases Th2/Th17-associated cytokines [296]. In A549 LC cells, fluoxetine reduces proliferation, migration, and colony formation and induces apoptosis, accompanied by decreased IDO1/IDO2 and increased caspases [296]. These findings suggest that fluoxetine may act through both stress-immunomodulatory and tumor-cell-intrinsic mechanisms in LC [296].

### Skin cancer

Evidence for stress-associated skin carcinogenesis is derived mainly from UV-induced mouse models. In SKH1 mice exposed to UVB, chronic restraint stress accelerated tumor emergence and progression and reduced spontaneous tumor regression. Mechanistically, stressed mice showed reduced cutaneous type 1 immune activity, including diminished IL-12- and IFN-γ-related responses, together with expansion of CD25^+^ suppressive T cell populations [135]. A complementary naturalistic predator-stress model also demonstrated earlier development of UV-induced cutaneous neoplasms in stressed mice than in nonstressed controls [7]. These findings support an immune-mediated interaction between chronic stress and UV injury; however, the evidence remains model-restricted and does not establish a general effect across melanoma or human skin cancers.

### Prostate cancer

In prostate cancer, chronic stress supports tumor survival and angiogenesis through adrenergic signaling. Stress-released epinephrine activates β2-AR and PKA, leading to inhibitory phosphorylation of the proapoptotic effector BAD and suppression of apoptosis; β-blockers, PKA inhibition, or nonphosphorylatable BAD variants such as S112A can interrupt this survival program [297]. In parallel, catecholamine stimulation of β2-AR signaling on tumor endothelium rewires endothelial metabolism toward a proangiogenic state, suggesting that β-blockade may dampen stress-responsive angiogenic programs in prostate cancer [298].

### Colon cancer

In colon cancer, the strongest stress-linked mechanism involves immune polarization. In BALB/c mice, prolonged aversive noise stress activates systemic stress responses, increases serum corticosterone and norepinephrine, and accelerates colon tumor growth [299]. Mechanistically, this stressor shifts helper T cell responses from a Th1 profile toward Th2 dominance: IFN-γ declines, whereas IL-4 and IL-10 increase [299,300]. This cytokine shift weakens cell-mediated antitumor immunity and increases susceptibility to colon cancer progression [299].

Additional stress-responsive programs may further promote colon cancer progression. Stress-induced TSC22D3-mediated suppression of dendritic-cell type I interferon signaling, as discussed in the Dendritic cells section, may represent an additional shared mechanism of immune evasion [184], while COX-2/PGE₂–VEGF-C remodeling promotes invasion, angiogenesis, and lymphatic spread [7]. These programs overlap with stress-responsive mechanisms reported in breast and lung cancer, suggesting that immune suppression and vascular/lymphatic remodeling represent shared outputs of chronic stress across multiple tumor contexts [7].

### Pancreatic cancer

Chronic stress promotes pancreatic cancer invasion mainly through a β2-AR-dependent invasive gene program. Catecholamines released under stress activate β2-AR signaling, elevate transcription of invasion-related genes, increase tumor-cell motility, promote penetration of adjacent tissue, and facilitate dissemination to distant organs [265]. Because this pathway depends on β2-AR, β-blockers may blunt this catecholamine-driven invasive program and serve as potential adjunctive agents in stress-associated pancreatic cancer progression [265,301].

### Lymphoma

In lymphoma models, chronic stress accelerates tumor growth, invasion, and metastatic capacity through both cancer-cell-intrinsic programs and immune-dependent mechanisms. In EL4 lymphoma, prolonged stress increases tumor growth and invasive capacity, while fluoxetine and sertraline attenuate these stress-driven effects [302]. Tumors from stressed animals show higher mRNA levels of cyclins A2, D1, and D3, together with reduced transcripts for the cyclin-dependent kinase inhibitors p15/Ink4b, p16/Ink4a, p21/Cip1, and p27/Kip1 [302]. These changes are consistent with faster cell-cycle progression and proliferative expansion [303–308].

Chronic stress also increases metastatic competence in lymphoma. Tumor cells derived from stressed animals produce more spontaneous metastases and form more metastatic nodules after transfer than tumor cells derived from control animals [302,309,310]. At the matrix-remodeling level, tumors from stressed animals show higher MMP-2 and MMP-9 and lower TIMP-1, TIMP-2, and TIMP-3 mRNA, accompanied by stronger directed migration in transwell assays [311,312]. Analyses of immune-depleted tumor-cell suspensions suggest that stress-driven tumor growth arises mainly from molecular changes in cancer cells, whereas increased invasive capacity reflects contributions from both malignant cells and infiltrating immune cells [302].

Immune mechanisms also appear central. Adoptive transfer experiments showed that animals receiving immune cells from stressed donors developed larger primary tumors and more spontaneous metastases than recipients receiving cells from control donors, even when the recipients themselves were not exposed to stress [302]. This indicates that, in this model, stress can accelerate lymphoma progression through immune cell-dependent mechanisms. Fluoxetine and sertraline mitigated these effects, likely by restoring antitumor immune function, although direct effects of stress mediators or selective serotonin reuptake inhibitors (SSRIs) on tumor cells cannot be excluded [313–321]. These findings support a model in which chronic stress drives lymphoma progression through combined cell-cycle acceleration, ECM remodeling, enhanced migration, and impaired antitumor immunity.

Overall, mechanistic support is strongest for breast, gastric, and lung cancer, where stress-responsive pathways have been linked to defined receptor axes, downstream signaling events, and intervention-sensitive phenotypes. Evidence in skin, prostate, colon, pancreatic cancer, and lymphoma remains more model-dependent and should be interpreted as mechanistically informative rather than clinically definitive.

## Chronic Stress and Therapeutic Implications

Because chronic stress persistently activates the SNS and the HPA axis, elevating catecholamines and GCs, it can engage β/α-adrenergic and GC-receptor programs across both tumor cells and multiple components of the TME. These stress-linked programs not only promote tumor progression but also can undermine the efficacy of radiotherapy, chemotherapy, targeted therapy, and immunotherapy by reshaping immune composition and function, reinforcing proangiogenic and inflammatory signaling, and facilitating adaptive resistance phenotypes within the TME. Accordingly, modulation of stress-responsive pathways has emerged as a rational adjunct to standard anticancer regimens. The major pharmacological and nonpharmacological strategies targeting stress-driven TME remodeling are summarized in Table 3. Pharmacologic strategies (e.g., β- and α-adrenergic antagonists) aim to blunt protumor neuroendocrine signaling and restore antitumor immune activity, while stress-reducing interventions seek to lower physiological stress load and sympathetic output, thereby improving the immunologic and stromal context in which anticancer therapies operate. The sections below, therefore, synthesize evidence for adrenergic blockade and related adjuncts, highlight combination signals across treatment modalities, and summarize nonpharmacological approaches as supportive strategies within an integrated “stress–cancer therapy” framework.

**Table 3.** Therapeutic strategies targeting chronic stress-driven tumor progression and tumor microenvironment remodeling.

Intervention category	Representative interventions	Primary targets/pathways	Core rationale in stress–cancer biology	Evidence type and maturity ^a^	References
β-Adrenergic blockade	Propranolol; metoprolol	β-AR; cAMP–PKA	Attenuates catecholamine-driven signaling, T cell dysfunction, myeloid suppression, angiogenesis, and treatment resistance	Level 3 for propranolol plus pembrolizumab in melanoma; level 4–5 for broader oncologic benefits	[189,322,331]
α1-Adrenergic blockade	Doxazosin; naftopidil	VEGF-mediated angiogenesis; tumor-cell growth/survival	May inhibit angiogenic responses and tumor-cell proliferation/survival in selected preclinical models	Level 5: predominantly preclinical evidence	[332,333]
GR antagonism	Mifepristone	GR signaling	May counter glucocorticoid-driven immune suppression, tumor-cell survival, and treatment resistance	Level 3–5: limited early clinical evidence with stronger mechanistic support	[354,355]
Stress-pathway combination therapy	β-Blocker plus COX-2 inhibitor, radiotherapy, chemotherapy, targeted therapy, or immunotherapy	β-AR, inflammatory, angiogenic, and resistance pathways	Aims to restore treatment sensitivity and improve antitumor immunity by disrupting stress-adaptive signaling	Level 3–5: early clinical, observational, and preclinical evidence depending on regimen	[324]
Microenvironment-targeting agents	Zoledronic acid; bevacizumab; tocilizumab; siltuximab	Myeloid remodeling; VEGF; IL-6	Targets stress-associated macrophage recruitment, vascular remodeling, and inflammatory signaling	Established general clinical uses, but level 5 evidence for stress-specific anticancer effects	[228,335–337]
Psychological and mind–body interventions	MBSR; CBT; yoga	HPA axis; sympathetic output; inflammatory signaling	Reduces perceived stress and improves quality of life; may favorably modulate neuroendocrine and immune parameters	Level 2 for patient-reported outcomes; level 4–5 for antitumor biological outcomes	[94,369]
Exercise and physiological stress reduction	Structured exercise; environmental enrichment; thermoneutral housing	Systemic inflammation; NK cells; CD8^+^ T cells; MDSCs	Reduces fatigue and stress burden and may improve immune fitness and checkpoint responsiveness	Level 2 for fatigue and quality-of-life outcomes; level 5 for stress-specific antitumor effects	[375–379]
Psychotropic and nutritional approaches	SSRIs; dietary modification; nutritional products	Central stress regulation; metabolic and inflammatory pathways	Primarily supports symptom control and general well-being; direct anticancer effects remain uncertain, and unsupervised supplement use may be harmful	Established supportive use in selected settings; level 4–5 for cancer-specific therapeutic benefit	[302,383–385]

β-AR, β-adrenergic receptor; cAMP, cyclic adenosine monophosphate; CBT, cognitive behavioral therapy; COX-2, cyclooxygenase-2; GR, glucocorticoid receptor; HPA, hypothalamic pituitary adrenal axis; IL-6, interleukin-6; MBSR, mindfulness-based stress reduction; MDSCs, myeloid-derived suppressor cells; NK, natural killer; PKA, protein kinase A; SSRIs, selective serotonin reuptake inhibitors; VEGF, vascular endothelial growth factor

^a^ Evidence maturity was categorized as follows: level 1, established clinical evidence or guideline-supported use; level 2, randomized clinical evidence; level 3, early-phase prospective clinical evidence; level 4, observational clinical evidence; and level 5, preclinical mechanistic evidence. Levels refer specifically to relevance in stress-associated cancer biology rather than to an intervention’s general clinical use.

### β-Blockers

β-Blockers inhibit catecholamine signaling at β-adrenergic receptors and thereby attenuate protumor β-AR-dependent pathways; representative agents include propranolol and metoprolol [322]. At the tumor–immune interface, β-AR blockade has been linked to mitigation of T cell exhaustion programs within the TME and to improved metabolic fitness and effector potential of TILs [189]. In stress-conditioned settings, propranolol has also been associated with reduced accumulation of immunosuppressive myeloid populations and with restrained tumor-promoting remodeling of the microenvironment [323]. Beyond immune effects, β-blockade has been reported to potentiate radiotherapy responses in certain tumor contexts, potentially through dampening inflammatory and proangiogenic signaling cascades [e.g., NF-κB–VEGF/epidermal growth factor receptor (EGFR)/COX-2] [324].

Combination strategies are increasingly explored. Preclinical studies support the potential benefit when β-blockers are paired with anti-inflammatory agents such as COX-2 inhibitors [89]. Early clinical observations further suggest that adding propranolol to standard anticancer regimens may improve patient-reported outcomes in some settings, and β-blocker co-medication has been examined as a modifier of outcomes in targeted-therapy cohorts [325,326]. In parallel, β-blockade has been positioned as an immunotherapy adjunct based on evidence that it can favorably reshape intratumoral immune composition and cytokine milieus and may enhance responses to checkpoint blockade or cancer vaccines in experimental systems [327,328]. Melanoma is a particularly relevant translational setting for this strategy because checkpoint blockade is an established treatment backbone and β-adrenergic signaling may modulate antitumor immune fitness. Retrospective melanoma data suggest that pan β-blocker use is associated with improved survival in patients receiving immunotherapy, but these findings remain hypothesis-generating because of confounding and treatment-selection bias [329,330]. In a phase I trial of treatment-naïve metastatic melanoma, propranolol plus pembrolizumab showed acceptable safety, no dose-limiting toxicities, and preliminary antitumor activity, with propranolol 30 mg twice daily selected as the recommended phase II dose [331].

### α-Blockers

Notably, α1-adrenergic antagonists have shown preclinical antitumor potential. Doxazosin inhibits endothelial-cell adhesion, migration, and invasion and suppresses VEGF-mediated angiogenic responses [332]. Naftopidil has likewise shown antiproliferative and cytotoxic effects in prostate cancer and other malignancies in preclinical studies [333]. Together, these findings warrant further evaluation of α1-adrenergic antagonists in selected cancer contexts.

### Stress-reducing interventions

Stress-targeted interventions can modulate sympathetic outflow and reshape the TME, thereby supporting anticancer therapy. Approaches that engage central reward circuitry can dampen sympathetic/β-adrenergic signaling and are associated with reduced accumulation of MDSCs in the TME [223]. Physiological and environmental strategies, such as thermal modulation (e.g., hyperthermia or thermoneutral housing) and environmental enrichment, have likewise been linked to a less immunosuppressive milieu, with lower MDSC burden and enhanced NK cell activity and intratumoral infiltration [323,334]. Consistent with such an immunologic shift, alleviating physiological stress has been reported to improve responsiveness to immune-checkpoint blockade in preclinical settings [195]. Clinically, stress-management programs such as yoga, mindfulness, and cognitive behavioral therapy (CBT) can improve patient-reported outcomes and have been associated with favorable immune signatures (e.g., increased proportions of antitumor immune subsets and supportive cytokine profiles), although available trials are generally small and follow-up is limited [94].

### Other therapeutic alternatives

Additional pharmacologic options may attenuate stress-associated remodeling of TME. Zoledronic acid has been reported to counter stress-linked myeloid remodeling, including reduced macrophage infiltration and modulation of PDGF-AA-related signaling [228]. Psychotropic agents are also being considered in this context, and SSRIs, such as fluoxetine and sertraline, can alleviate chronic stress-related symptoms and have been explored as adjuncts in oncology, although stronger clinical evidence is still required to define anticancer benefit, appropriate populations, and optimal combinations [302]. Another complementary approach is to target stress-inducible inflammatory and vascular pathways. Bevacizumab (anti-VEGF) is approved for multiple solid tumors and is commonly deployed in combination regimens [e.g., with chemotherapy and, in selected settings, immune-checkpoint blockade, anti-EGFR therapy, or poly(adenosine diphosphate-ribose) polymerase (PARP) inhibitors] [335]. By contrast, IL-6-axis blockade (e.g., tocilizumab and siltuximab) has shown variable and context-dependent antitumor effects in oncology studies, while tocilizumab remains an established intervention for cytokine-release syndrome following chimeric antigen receptor (CAR) T cell therapy [336,337].

### Pharmacological treatment: Overall evidence and caveats

Chronic stress predominantly signals through adrenergic and GR pathways. β-Adrenergic blockers (BBs) interrupt catecholamine binding to β-adrenergic receptors and have been associated with improvements in quality of life as well as reductions in anxiety and depressive symptoms in patients with cancer. Observational studies further suggest associations between BB use and reduced risks of distant metastasis, recurrence, and cancer-specific mortality across several tumor types [57,60,338–342]. In clinical practice, BBs are also routinely administered as antihypertensive agents to mitigate chemotherapy-related cardiotoxicity [343].

Despite these encouraging signals, evidence for a consistent survival benefit remains inconclusive. Retrospective analyses report heterogeneous outcomes for overall and progression-free survival, with effects that appear to vary by tumor type, disease stage, and the pharmacologic properties of individual BBs. Moreover, any potential benefit may be confounded or attenuated by coexisting psychological conditions, including anxiety and depression, which themselves influence prognosis and treatment tolerance [344–353]. In addition, activation of GR signaling can partially counteract β-adrenergic blockade, potentially limiting the efficacy of β-AR-focused strategies in stress-exposed settings [184].

Direct targeting of GR signaling has, to date, shown limited anticancer efficacy as a monotherapy. For example, the GR antagonist mifepristone has not demonstrated robust single-agent antitumor activity in BC, although low-dose regimens have been reported to improve quality of life and reduce leiomyoma burden in non-oncologic contexts [354,355]. More broadly, neuromodulatory agents face inherent limitations, including restricted tumor specificity and the risk of systemic adverse effects at doses required to influence tumor biology. Collectively, these considerations underscore the need for well-designed randomized controlled trials to delineate patient subgroups, rational combination strategies, and dosing paradigms in which modulation of stress-responsive pathways can deliver meaningful clinical benefit.

### Combating treatment resistance by disrupting stress adaptation

Chronic stress can compromise the efficacy of anticancer therapies by activating β-adrenergic signaling and GC pathways that collectively suppress apoptosis and promote resistance to radiotherapy and chemotherapy. Sustained stress elevates endogenous GCs, which have been shown to attenuate cytotoxic responses and diminish treatment sensitivity across multiple tumor contexts. Preclinical evidence indicates that GC exposure enhances ECM adhesion and reduces responsiveness to platinum- and taxane-based chemotherapy, while stress conditioning can broadly blunt the effectiveness of DNA-damaging regimens [184,238]. Consistent with these findings, high intratumoral GR expression has been associated with inferior outcomes in patients receiving chemotherapy, suggesting a clinically relevant role for GR signaling in therapy resistance [79].

Catecholamine signaling further contributes to stress-associated treatment failure. Endogenous norepinephrine and epinephrine have been reported to impair p53 signaling, increase genomic instability, and reduce chemotherapy-induced apoptosis, thereby weakening drug efficacy [236,356]. Importantly, modulation of stress-responsive signaling has been associated with improved therapeutic responses in clinical settings, supporting the translational relevance of targeting these pathways [357].

Beyond cytotoxic therapies, chronic stress also interferes with anti-angiogenic and molecularly targeted treatments. Stress-induced β-adrenergic–cAMP–PKA signaling enhances VEGF expression within the TME, undermining VEGF-directed strategies and reducing the efficacy of anti-angiogenic agents in preclinical models [80,358]. Catecholamine signaling has likewise been implicated in resistance to targeted therapies, including EGFR inhibition, through mechanisms involving connexin regulation and altered MET/IGF-1R signaling [16]. In non-small cell lung cancer, β₂-adrenergic activation disrupts LKB1 signaling and amplifies IL-6 and MAPK pathway activity, promoting resistance to EGFR tyrosine kinase inhibitors [326].

In line with these mechanistic insights, clinical and translational studies increasingly explore stress-modulating strategies as adjuncts to standard cancer therapy. The addition of BBs or GR antagonists to radiotherapy or chemotherapy has been reported to delay resistance and prolong progression-free survival in selected patient populations [326,359]. Retrospective cohort analyses further suggest that β-blocker co-therapy, when combined with anti-angiogenic agents, immunotherapy, radiotherapy, or chemotherapy, is associated with improved overall survival, although prospective validation remains necessary [58,63,64,330,331,360,361].

### Combining stress-targeted approaches with immunotherapy

Chronic stress has been shown to impair antitumor immunity by reducing CD8^+^ T cell abundance and compromising CTL function, thereby undermining the efficacy of cancer vaccines and immunotherapies [186]. A central mediator of this effect is endogenous GC signaling. Activation of the GR in CD8^+^ TILs promotes transcriptional programs associated with T cell exhaustion and has been linked to poor responses to immune-checkpoint blockade in both preclinical systems and patients with melanoma [182]. Consistent with this framework, stress exposure skews the intratumoral immune landscape toward immunosuppression, characterized by increased Tregs and reduced infiltration of effector CD8^+^ lymphocytes, a pattern that correlates with diminished responsiveness to PD-1/PD-L1-targeted therapies [184,362].

Mechanistically, stress-inducible transcriptional regulators further reinforce immune dysfunction. The GC-responsive factor TSC22D3 has been identified as a key suppressor of antitumor immunity, limiting the effectiveness of both prophylactic cancer vaccination and PD-1–based immunotherapy [184]. In parallel, psychological stress has been associated with reduced expression of IL-2 receptors on peripheral leukocytes, providing a plausible explanation for impaired responses to IL-2-based immunotherapeutic strategies [363].

Emerging evidence suggests that pharmacologic modulation of stress signaling may partially restore antitumor immune competence. In preclinical studies, β-adrenergic blockade counteracts stress-driven immune remodeling, enhances CD8^+^ T cell activity, and improves responses to cytotoxic and immunotherapeutic interventions [195]. Complementing these findings, retrospective data in metastatic melanoma associate concomitant pan β-blocker use with improved overall survival among patients receiving immunotherapy, while an early phase I trial of propranolol plus pembrolizumab demonstrated acceptable safety and preliminary antitumor activity [330,331].

Together, these observations support a model in which chronic stress compromises immunotherapy efficacy through coordinated GC- and adrenergic-mediated immune suppression. Given that many neurotropic agents are already clinically approved, a rational combination of stress-modulating drugs with immune-checkpoint blockade, cancer vaccines, or cytotoxic therapies represents a feasible and clinically actionable strategy to enhance antitumor immunity, warranting further prospective evaluation [364].

### Nonpharmacological treatment

A broad range of complementary and integrative interventions has been explored in oncology with the aim of reducing stress burden and improving patient-reported outcomes. When implemented alongside standard surgical and pharmacological treatments, these approaches are primarily associated with improvements in quality of life, mitigation of treatment-related symptoms, and, in some contexts, modulation of immune and neuroendocrine parameters relevant to tumor progression [365]. Within this nonpharmacological domain, psychological interventions, structured physical activity, and dietary or supplement-based strategies represent the most extensively studied modalities. However, the robustness and clinical generalizability of the available evidence remain heterogeneous, varying substantially across cancer types, disease stages, and intervention designs.

#### Psychological interventions

Multiple psychological interventions have been shown to reduce perceived stress and enhance social support among patients with cancer [366,367]. Among these, mindfulness-based approaches emphasize present-moment awareness and stress regulation, with the goal of alleviating anxiety related to diagnosis, treatment, and fear of recurrence while providing sustained psychological support [368]. Across interventional studies, mindfulness-based stress reduction (MBSR) programs are consistently associated with improvements in quality of life and reductions in stress- and mood-related symptoms in patients with cancer, particularly in BC cohorts [369]. Notably, these patient-reported benefits have been accompanied by measurable physiological correlates, including attenuation of HPA axis activity, reduced proinflammatory cytokine levels, and partial restoration of NK cell activity, suggesting potential links between psychological stress modulation and immune regulation [370,371]. Randomized trials further support the beneficial effects of mindfulness-based interventions on cancer-related fatigue, sleep disturbance, and selected biological health markers [372,373]. However, as the majority of available evidence derives from studies in BC populations, the extent to which these findings generalize to other malignancies and disease contexts remains to be established.

#### Exercise

Alongside psychological interventions, structured physical activity during and after cancer therapy has been associated with improvements in survival and recurrence-related outcomes in some settings, while its effects on psychosocial well-being, cognition, and depressive symptoms remain an active area of investigation [374]. Across randomized and controlled studies, exercise interventions are consistently linked to reductions in cancer-related fatigue during treatment [375–377]. Beyond symptom relief, physical activity has also been associated with favorable immunometabolic shifts, including attenuation of systemic inflammatory markers, enhanced CD8^+^ T cell activity, and partial restoration of an immune milieu supportive of antitumor responses [378,379].

Within this spectrum, mind–body exercise modalities such as yoga are widely adopted by patients, particularly in BC populations, and are generally associated with improvements in fatigue, mood, and quality of life, as well as with favorable shifts in inflammatory profiles [380–382]. Collectively, current evidence supports structured physical activity as a feasible and low-risk strategy to mitigate stress burden, fatigue, and depressive symptoms in people with cancer. At the same time, the reported outcomes fall into 2 distinct domains—patient-reported symptom relief and quality of life (e.g., fatigue, mood, and sleep) versus biological effects on stress signaling, immune function, and treatment response—with a more mature evidence base for the former, leaving the biological effects comparatively underdetermined. Because the available data are also dominated by short-term studies in selected tumor types, future behavioral and exercise trials should be larger and longer in duration, span diverse malignancies, and pre-specify a focused panel of mechanistic and treatment-response endpoints. Priority should be given to diurnal cortisol rhythm (stress signaling); circulating inflammatory cytokines together with NK and CD8^+^ T cell activity (immune function); and treatment-response endpoints such as chemotherapy completion and response to immune checkpoint blockade, to establish whether symptom benefits are accompanied by durable biological and survival advantages.

#### Dietary strategies, herbs, and nutritional products

Dietary patterns can modulate metabolic, inflammatory, and neuroendocrine pathways implicated in both cancer development and stress-related disorders. Observational studies have associated traditional dietary patterns, such as Mediterranean, Norwegian, and Japanese diets, as well as higher consumption of fruits, vegetables, whole grains, nuts, and seeds, with favorable cancer-related outcomes and reduced depressive symptoms [383–385]. In parallel, randomized trials conducted in depression cohorts indicate that hypocaloric dietary interventions, particularly when combined with physical activity, can improve mental and physical health parameters, providing a conceptual rationale for integrated lifestyle approaches in stress-burdened populations [386,387].

Within complementary strategies, vitamins and herbal products are among the most frequently used interventions by patients with cancer [387]. Certain supplements have been reported to alleviate stress-related symptoms such as anxiety or fatigue in selected settings; however, the evidence base remains heterogeneous and largely context-dependent [388]. Importantly, accumulating data indicate that unsupervised supplement use may interfere with oncologic therapies. Observational analyses have linked antioxidant supplementation during treatment (including vitamins A, C, E, or coenzyme Q10) with higher risks of cancer recurrence and mortality, and iron or vitamin B12 use before or during chemotherapy has likewise been associated with adverse outcomes [389].

Taken together, these findings underscore that while dietary quality and lifestyle-oriented nutritional strategies may support stress reduction and overall well-being in patients with cancer, indiscriminate use of vitamin or herbal supplements carries potential risks. Careful clinical guidance and tumor- and treatment-specific considerations are therefore essential when incorporating nutritional products into cancer care, particularly during active therapy.

## Challenges and Future Directions

Translating stress–cancer biology into patient benefit faces several immediate obstacles. Adrenergic signaling is heterogeneous across tumor and stromal compartments, with β1-, β2-, and β3-adrenergic receptors contributing in context-dependent ways, and accordingly, some settings may favor nonselective β-blockade over β1-selective agents. Presynaptic α2 autoreceptors further fine-tune norepinephrine release, generating effects of α-adrenergic modulation that are notably state-dependent and occasionally opposing, which complicates combination therapy design. Systemic β-blockade also blurs attribution of efficacy to tumor cell-intrinsic versus stromal, vascular, and immune mechanisms, slowing the development of precise biomarkers. At the population level, self-reported stress exposure is noisy and susceptible to bias, limiting causal inference unless triangulated with objective neuroendocrine readouts and mechanistic models. Microbiota–metabolite pathways, including the Blautia–acetate–CD8^+^ T cell axis, remain difficult to validate clinically because many cohorts lack paired stool profiling and immune/TME measurements. Finally, signals of benefit from β-blockers and related strategies often appear tumor-type specific, and much of the available clinical evidence still relies on small cohorts or short follow-up.

**Fig. 6.**
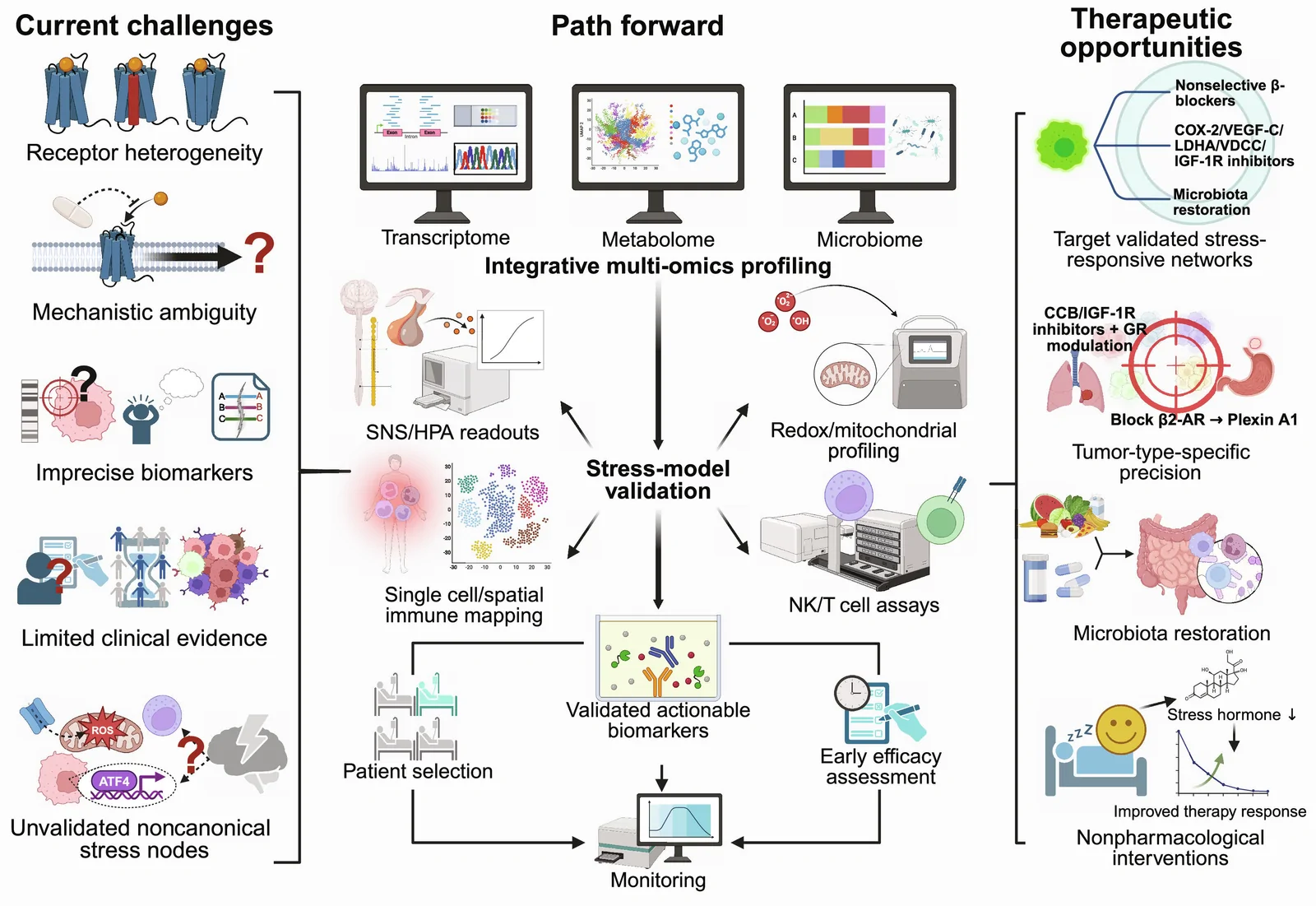
Key challenges and future directions for translating chronic stress-cancer biology into precision therapeutics. This schematic summarizes major barriers and an actionable path forward for developing stress-modulating strategies in oncology. Left panel (Current challenges): Stress-responsive signaling is context-dependent, with β1-, β2-, β3-, and presynaptic α2-receptor functions varying across tumor, stromal, vascular, and immune compartments, complicating agent selection and prediction of heterogeneous responses. Systemic β-blocker effects are often multifactorial, making it difficult to distinguish tumor cell-intrinsic from immune, vascular, and stromal mechanisms. Biomarker frameworks remain imprecise because stress exposure is frequently captured by noisy self-report measures, paired multi-omics datasets are limited, microbiota–metabolite–immune axes require clinical validation, and existing clinical evidence often derives from small cohorts, short follow-up, or tumor-type-specific signals. Middle panel (Path forward): Integrative transcriptomic, metabolomic, microbiome, redox/mitochondrial, and immune-profiling approaches should be coupled with objective SNS/HPA readouts and tumor-bearing chronic stress models. This validation layer can test candidate noncanonical stress-sensing nodes, including TRPM8–ROS and ISR–ATF4-related programs, without assuming direct chronic stress causality. Validated and traceable biomarkers can then support rational patient selection, early efficacy assessment, and longitudinal monitoring of stress signaling and its reversal during therapy. Right panel (Therapeutic opportunities): Biomarker-guided stratification may enable the targeting of validated stress-responsive networks. Potential strategies include nonselective β-blockade; rational combination approaches directed at COX-2, VEGF-C, LDHA, the VDCC–IGF-1R axis, or GR in selected contexts; microbiota-based restoration approaches; tumor-type-specific pathway targeting; and nonpharmacological interventions aimed at reducing physiological stress load. This figure was generated with BioRender (https://biorender.com). UMAP, uniform manifold approximation and projection; COX-2, cyclooxygenase-2; VEGF-C, vascular endothelial growth factor C; LDHA, lactate dehydrogenase A; VDCC, voltage-dependent calcium channel; IGF-1R, insulin-like growth factor 1 receptor; CCB, calcium channel blocker; GR, glucocorticoid receptor; β2-AR, β2-adrenergic receptor; ROS, reactive oxygen species; ATF4, activating transcription factor 4; SNS, sympathetic nervous system; HPA, hypothalamic pituitary adrenal axis; NK cells, natural killer cells; T cells, T lymphocytes.

Future studies should directly test whether noncanonical tumor-cell stress-sensing nodes are engaged by chronic psychological or neuroendocrine stress in tumor-bearing models (Fig. 6). Although melanoma data indicate that TRPM8 modulation can induce mitochondrial ROS-dependent apoptotic priming and increase ULBP1/NKG2D-mediated NKCC, this evidence does not establish chronic stress causality [42]. Therefore, models combining defined stress paradigms with SNS/HPA readouts, tumor redox and mitochondrial profiling, and single-cell or spatial immune mapping are needed. Parallel assessment of ISR–ATF4 activation, NK and T cell functional assays, and ligand–receptor analyses would clarify whether ROS, mitochondrial stress, and ion-channel signaling represent stress-driven immune vulnerabilities or context-specific pharmacologic effects [41].

A clear path forward is to use integrative multi-omics and systems biology to convert mechanisms into measurable, actionable markers (Fig. 6). Building on the transcriptomic, single-cell/spatial, and stable-isotope tracing data linking acetate availability to CD8^+^ T cell function and metabolic flux, real-world studies should incorporate fecal microbiome profiling together with circulating and intratumoral metabolite measurements. An integrated analysis of these layers, including transcriptional state, metabolic flux, microbiome composition, and metabolite exposure, will clarify the competition for and signaling through acetate between tumor cells and T cells. This knowledge is expected to yield tractable biomarkers for patient stratification, pharmacodynamic monitoring, and early efficacy assessment. For the *Blautia*–acetate–CD8^+^ T cell axis specifically, this translates into a small set of clinically measurable readouts: fecal *Blautia* relative abundance (16S ribosomal RNA or shotgun metagenomic sequencing), serum and fecal acetate (short-chain fatty acid quantification by mass spectrometry), and intratumoral CD8^+^ T cell density and IFN-γ production (multiplex immunohistochemistry or flow cytometry); the CD8^+^ T cell acetyl-CoA pool can serve as a complementary, mechanism-level readout of acetate utilization where tissue permits [286].

Such biomarkers can, in turn, guide mechanism-based combination designs that directly address stress-responsive networks. Rational partners include nonselective β-blockers, COX-2 or VEGF-C inhibition, LDHA-directed approaches (including pharmacologic ascorbate), calcium-channel or IGF-1R blockade, and microbiota-based acetate restoration. These strategies should be evaluated alongside surgery, chemotherapy, radiotherapy, targeted therapy, and immunotherapy in prospectively designed, biomarker-stratified trials. This axis is a high-priority candidate for such trials because it links a modifiable, low-risk input (dietary fiber, acetate-producing probiotics such as *Blautia*, or acetate supplementation) to a quantifiable circulating mediator (acetate) and a measurable antitumor output (CD8^+^ T cell infiltration and IFN-γ), and because it is one of the few stress–microbiome links supported by both causal preclinical rescue experiments and concordant human correlative data [286]. The same markers map directly onto trial roles: baseline serum acetate and fecal *Blautia* abundance as stratification or enrichment criteria (selecting acetate-low, CD8-low patients), acetate- or fiber-based microbiota restoration as an adjunct arm to immune-checkpoint blockade, and serial serum acetate with on-treatment intratumoral CD8^+^ density and IFN-γ as paired pharmacodynamic and early-efficacy endpoints. This rationale parallels human and preclinical evidence that fiber-driven, short-chain fatty acid-producing gut microbiota are associated with improved checkpoint-blockade responses in patients and with higher intratumoral IFN-γ^+^ cytotoxic T cell frequencies in preclinical models [390]. This calls for a tailored modulation of stress pathways, guided by the patient’s unique receptor programs, metabolic dependencies, and immune-microbiome features, rather than a uniform, one-size-fits-all intervention.

Organ- and pathway-specific precision offers additional leverage. In gastric cancer, stress-linked β2-AR signaling has been reported to engage downstream circuits (including PlexinA1-associated programs) that promote proliferation and EMT, nominating the epinephrine/β2-AR axis as a testable intervention node. In LC, β-AR–PKA–VDCC signaling can couple to IGF-1R activation, while GR–TSC22D3-mediated type I interferon dampening has been implicated in immune evasion; selected populations could therefore be explored for calcium-channel blockers and/or IGF-1R inhibitors as preventive or therapeutic partners, and for strategies that counter GR-linked immunosuppressive programs. These tumor-tailored hypotheses illustrate how stress circuitry can be mapped to concrete, pathway-level therapies.

Behavioral and physiological stress-reduction strategies also warrant rigorous integration with oncologic care (Fig. 6). By dampening excessive adrenergic and inflammatory tone, psychological interventions and physiological modulation may improve treatment tolerance and potentially enhance responses to systemic therapy. Definitive tests will require adequately powered randomized studies with durable survival and immune-quality endpoints, alongside embedded biomarker programs that quantify stress signaling and its reversal.

## Conclusion

Chronic stress emerges as a systems-level modifier of cancer biology that couples sustained neuroendocrine activation to coordinated tumor-intrinsic, metabolic, and microenvironmental reprogramming. Persistent engagement of the SNS and HPA axis maintains elevated catecholamines and GCs, which exert broad systemic effects while also signaling directly through adrenergic receptors and the GR across malignant, stromal, vascular, and immune compartments. Within tumors, these inputs converge on prosurvival and therapy-resistance programs, promote angiogenic and matrix-remodeling cues, and facilitate metastatic dissemination, thereby providing a mechanistic bridge between psychosocial stress exposure and clinically relevant cancer phenotypes.

A central theme of this review is that stress signaling remodels the TME as an integrated ecosystem rather than acting solely on tumor cells. Adrenergic and GC pathways directly suppress antitumor immunity by impairing dendritic-cell maturation and antigen presentation, weakening NK cell cytotoxicity, and promoting CD8^+^ T cell dysfunction and exhaustion while concurrently expanding immunosuppressive myeloid circuits and stress-responsive inflammatory mediators. In parallel, stress-associated cytokine programs, VEGF-driven vascular remodeling, EMT, and ECM degradation reduce both physical and immunologic barriers to invasion and dissemination. These interlocking effects help explain why chronic stress may exert modest effects on primary tumor growth in some contexts yet markedly amplify metastatic spread and therapy resistance in others.

Translationally, these insights provide a strong rationale for adjunct strategies that attenuate stress-conditioned tumor programs, including β- and α-adrenergic antagonism, GR-modulating approaches, and behavioral stress-reduction interventions integrated with standard cancer therapies. However, moving from association to actionable causality in patients will require biomarker frameworks capable of quantifying stress signaling and its modulation, disentangling tumor-intrinsic versus stromal and immune contributions, and accounting for tumor-type-specific and context-dependent pathway dominance. Integrative multi-omics approaches that link transcriptional state, metabolic exposure, immune composition, and microbiome features offer a promising route to define stress-responsive phenotypes, enable patient stratification, and guide mechanism-based combination regimens.
